# Predicted regulatory SNPs reveal potential drug targets and novel companion diagnostics in psoriasis

**DOI:** 10.1016/j.jtauto.2021.100096

**Published:** 2021-04-05

**Authors:** Andrea Virginia Ruiz Ramírez, Adolfo Flores-Saiffe Farías, Rocío del Carmen Chávez Álvarez, Ernesto Prado Montes de Oca

**Affiliations:** a Laboratory of Regulatory SNPs, Personalized Medicine National Laboratory (LAMPER), Medical and Pharmaceutical Biotechnology, Research Center of Technology and Design Assistance of Jalisco State (CIATEJ A.C.), National Council of Science and Technology (CONACYT), C.P. 44270, Guadalajara, Jalisco, Mexico; b Doctorate Program in Human Genetics, Health Sciences Campus (CUCS), Guadalajara University, Sierra Mojada 950, Col. Independencia, C.P. 44340, Guadalajara, Jalisco, Mexico; c Laboratory of Pharmacogenomics and Preventive Medicine, LAMPER, Pharmaceutical and Medical Biotechnology, CIATEJ, A.C., CONACYT, C.P. 44270, Guadalajara, Jalisco, Mexico; d Scripps Research Translational Institute, 3344 North Torrey Pines Court, Suite 300, La Jolla, CA, 92037, USA; e Integrative Structural and Computational Biology, Scripps Research Institute, 10550 North Torrey Pines Road, SGM 300, La Jolla, CA, 92037, USA

**Keywords:** Psoriasis, Bioinformatics, Gene expression regulation, Single nucleotide polymorphism, Regulatory SNPs, Transcription factors, Interleukin-17, Antimicrobial cationic peptides, Tumor necrosis factor, Cathelicidin, Etanercept, Secukinumab, Brodalumab

## Abstract

Psoriasis is an autoimmune disease associated with interleukins, their receptors, key transcription factors and more recently, antimicrobial peptides (AMPs). Cathelicidin LL-37 is an AMP proposed to play a fundamental role in psoriasis etiology. With our proprietary software SNPClinic v.1.0, we analyzed 203 common SNPs (MAF frequency ​> ​1%) in proximal promoters of 22 genes associated with psoriasis. These include nine genes which protein products are classic drug targets for psoriasis (*TNF, IL17A, IL17B, IL17C, IL17F, IL1*7RA*, IL12A, IL12B* and *IL23A*). SNPClinic predictions were run with DNAseI-HUP chromatin accessibility data in eight psoriasis/epithelia-relevant cell lines from ENCODE including keratinocytes (NHEK), T_H_1 and T_H_17 lymphocytes. Results were ranked quantitatively by transcriptional relevance according to our novel Functional Impact Factor (FIF) parameter. We found six rSNPs in five genes (*CAMP*/cathelicidin, *S100A7/*psoriasin*, IL17C, IL1*7RA and *TNF*) and each was confirmed as true rSNP in at least one public eQTL database including GTEx portal and ENCODE (Phase 3). Predicted regulatory SNPs in cathelicidin, *IL17C* and *IL1*7RA genes may explain hyperproliferation of keratinocytes. Predicted rSNPs in psoriasin, *IL17C* and cathelicidin may contribute to activation and polarization of lymphocytes. Predicted rSNPs in *TNF* gene are concordant with the epithelium-mesenchymal transition. In spite that these results must be validated *in vitro* and *in vivo* with a functional genomics approach, we propose FOXP2, RUNX2, NR2F1, ELF1 and HESX1 transcription factors (those with the highest FIF on each gene) as novel drug targets for psoriasis. Furthermore, four out of six rSNPs uncovered by SNPClinic v.1.0 software, could also be validated in the clinic as companion diagnostics/pharmacogenetics assays for psoriasis prescribed drugs that block TNF-α (e.g. Etanercept), IL-17 (e.g. Secukinumab) and IL-17 receptor (Brodalumab).

## Highlights

• We found six putative regulatory SNPs in cathelicidin (LL-37), psoriasin (*S100A7*), *IL17C, IL1*7RA and *TNF* genes.
• These rSNPs could be validated also as companion diagnostics/pharmacogenetics assays for most approved psoriasis drugs.
• Regulatory SNPs in *TNF* gene are concordant with the epithelial-mesenchymal transition.
• Regulatory SNPs in *IL17C* and *IL1*7RA may partially explain hyperproliferation of keratinocytes.
• Regulatory SNP rs12049559 in psoriasin (*S100A7*) may contribute to T-cell polarization.

## 1 Introduction

High-throughput techniques have facilitated to annotate and analyze human genetic variations such as single nucleotide polymorphisms (SNPs) [1]. Several studies have experimentally proved that SNPs within transcription factor binding sites (TFBSs) or other regulatory regions, called rSNPs, can modify (increase or decrease) the transcription rate of target genes by modifying the transcription factor (TF) affinity to DNA and thus, cause disease or susceptibility to disease phenotype [2,3]. However, the correct *in silico* prediction of rSNPs and the identification of the TF associated to the regulatory region has been a challenge due to the many factors involved in gene expression [4]. GWAS and other association studies have statistically associated SNPs with certain disease, however they do not describe the biological mechanism of the association and does not describe if the SNPs is functional or not [5]. Furthermore, GWAS do not provide a deep understanding of the relationship between the SNP and the disease. One step ahead will be to discover rSNPs in psoriasis if the *in silico* tools provide information about the transcription factors affected on their binding site. This will allow us to propose those TFs as novel therapeutic targets and those rSNPs as potential companion diagnostics.

Psoriasis is a chronic inflammatory disease caused by an exacerbated immune system response [6,7] that affects around 2–3% of the worldwide population [8]. It is characterized by red and scaly skin lesions due to an hyperproliferation of epidermal keratinocytes [8]. It involves genetic risk factors and environment triggers. As for the genetic risk factors, it has been found that several immune genes have a role in psoriasis whose biological mechanisms in the disease have not been yet fully understood. There is the possibility that alleles of this kind of genes decrease the threshold for the activation of the innate immune response [7]. It has been considered an autoimmune chronic disease of keratinocytes characterized by an increased number of immune cells (mainly T Lymphocytes and dendritic cells in dermis as well as neutrophils in epidermis) and activated immune pathways within psoriatic skin lesions [[9], [10], [11], [12]]. The pathogenesis of the disease is still unknown. However, some studies have shown T_reg_ lymphocytes are dysfunctional in psoriasis, with decreased suppressive capacity, suggesting that psoriasis may result from the inability to suppress autoinflammation [13]. Also, recent discoveries have found associations between innate antimicrobial peptides (AMPs), that are small molecules with multiple functions including chemotactic properties. In the present study we include the analysis of protein coding genes of three families of AMPs with an important role in psoriasis: cathelicidin, S100 proteins (including psoriasin) and defensins [6], as well as another psoriasis-related genes involved in T-cell development, T-cell polarization, inflammatory processes and classic therapeutic targets.

We applied our software SNPClinic to 22 psoriasis-related genes. In order to have a more specific search of the TFBSs that could be affected by the rSNPs in psoriasis disease, we focused our analysis in cell lines and tissues implicated in skin inflammation from the Encyclopedia of DNA Elements (ENCODE) Project.

SNPClinic could be used to efficiently identify rSNPs in promoter regions and the transcription factor binding sites (TFBSs) disturbed by the SNP. This information could be useful to discover transcription factors as novel drug targets and rSNPs that could be co-developed as companion diagnostics.

## 2 Materials and methods

### 2.1 Input data for the SNPClinic v.1.0

We generated *in silico* human proximal (2 ​Kb from transcriptional start site) pseudo-promoters comprising all common SNPs (MAF >1%) in the averaged world population (code=ALL) according to the 1000 Genomes Project for each of the 22 psoriasis genes, including transcription factors, antimicrobial peptides/proteins and interleukins and their receptors (Table 1). Then, to assess chromatin accessibility, we obtained DNAseI- HUP data from eight psoriasis/epithelia relevant cell lines from ENCODE project, namely normal human epithelial keratinocytes (NHEK), melanocytes (MELANO), monocytes (MONOCYTESCD14RO01746), human amniotic epithelial cells (HAEpiC), naïve T lymphocytes (T_H_0), helper T lymphocytes 1 (T_H_1), helper T lymphocytes 2 (T_H_2) and helper T lymphocytes 17 (T_H_17). Transcription factor Position Frequency Matrices (PFMs) were obtained from JASPAR database [14]. Thus we obtained in this step chromosome coordinates, biallelic alleles, DNAse-HUP accessibilities and PFMs as input data. SNPClinic v.1. was updated from MatLab (β-version in [15]) to Python.

**Table 1:** List of the psoriasis genes and their total number of common SNPs (>1%) in proximal promoters according to “ALL population” from 1000 Genomes Project [49] that were analyzed with SNPClinic v.1.0 software (β-version published in Ref. [15]).

Class	Gene	Common SNPs (MAF >1%)
*Interleukins/receptors*	*TNF*	*14*
	*IFNG*	*9*
	*IL12A*	*7*
	*IL12B*	*1*
	*IL17A*	*8*
	*IL17F*	*8*
	*IL1*7RA	*10*
	*IL17C*	*12*
	*IL19*	*6*
	*IL20*	*7*
	*IL22*	*14*
	*IL23A*	*2*
	*IL36A*	*5*
*Antimicrobial peptides/proteins*	*CAMP*	*3*
	*S100A7*	*9*
	*S100A7A*	*10*
	*S100A8*	*9*
	*S100A9*	*12*
	*DEFB1*	*32*
	*DEFB4*	*4*
*Transcription factors*	*STAT1*	*18*
	*NFKB1*	*3*
*Total*	22 genes	203 SNPs

### 2.2 Regulatory SNP prediction with SNPClinic v.1.0

We followed the method of Flores Saiffe et al., [15]. Briefly, with the data from subsection 2.1, SNPClinic scans for chromatin accessibility overlapping between DNAse HUP-1 of psoriasis/epithelia relevant cell line and common SNPs. If the chromatin of SNP is accessible, two Transcription Factor Relative Binding Score (RBS) are calculated, the RBS for the Major Allele (RBS_M_) and RBS for the Minor Allele (RBS_m_). SNPClinic scans base-per-base the proximal promoter for each gene for each of the two DNA strands calculating the local DNA affinity to each of the 396 human transcription factors from JASPAR Database [14]. The outputs of SNP Clinic v.1.0 are exact TF binding sequence, TFBS strand (coding/+, non-coding/-), altered TFBS, RBS_M,_ RBS_m,_ affinity impact (%), homotypic redundance (HR), homotypic redundance weight factor (HWF) [15] and our novel functional impact factor (FIF).

### 2.3 Functional interpretation of putative rSNPs

The hypothesis for genomic interpretation of data obtained in subsection 2.2 were generated reviewing Reactome Database [48], Ensembl [49] Expression Atlas [50], GTEx databases [51], OMIM [52], KEGG [53] and PubMed [54].

## 3 Results and discussion

In this work we applied the software SNPClinic v.1.0, useful in the identification of altered transcription factor binding sites (TFBSs) by the presence of a certain regulatory SNP (rSNP). Our group has demonstrated the utility of SNPClinic in the prediction of rSNPs to dissect comorbidities [15],in the prediction of altered constitutive gene expression by rSNPs [18] and in the pathogenic prediction of non-classical mutations in cancer [55].

The selection and ranking of the TF-SNP association is mainly based on the calculated percentage of the binding affinity impact taking in account also the homotypic redundancy which is useful in the determination of the possible impact that a polymorphism could have in the activation or repression of a gene, unlike other less sensitive methods which are mainly based on *p*-value filtering and/or ranking. The obtained results suggest that statistical ranking methods alone are not sufficient to predict correctly, because they should integrate as much biological context as possible to obtain relevant biological results, such as the chromatin accessibility, cell line/tissue specificity and the DNA sequence itself.

We applied our software SNPClinic to 22 psoriasis-related genes (Table 1) to analyze putative genetic variations within proximal promoters that could participate in psoriasis development (Table 2 and Fig. 1). Those genes were selected both from PubMed [54] and from the OMIM [52]. The five genes with the presence of putative rSNPs within an accessible region were *CAMP, IL17C, IL1*7*RA,* *S100A7* and *TNF* (Table 2 and Fig. 1). The six uncovered rSNPs by SNPClinic were confirmed as true eQTLs in at least one out of four public databases (Table 3 and Fig. 2, Fig. 3, Fig. 4, Fig. 5, Fig. 6, Fig. 7, Fig. 8, Fig. 9, Fig. 10, Fig. 11, Fig. 12, Fig. 13, Fig. 14, Fig. 15, Fig. 16, Fig. 17, Fig. 18, Fig. 19, Fig. 20, Fig. 21, Fig. 22, Fig. 23, Fig. 24, Fig. 25, Fig. 26, Fig. 27, Fig. 28, Fig. 29).

**Table 2:** Prediction of rSNPs with SNPClinic v.1.0 software. SNPClinic outputs include cell line specificity, altered TFBS and quantitative ranking according to transcriptional relevance with our novel Functional Impact Factor (FIF, see Material and Methods, section 2).

Gene	SNP	Chromatin-accesible cell line	Transcription factor	RBS_M_	RBS_m_	Affinity impact %	HR	HWF	FIF^a^
*CAMP*	rs9844566 (C/G)	NHEK	FOXP2	0.81	0.68	−15.34	1	1	−15.34
			TFCP2	0.88	0.76	−13.77	1	1	−13.77
			ARNT::HIF1A	0.80	0.63	−20.79	2	0.5	−10.39
			ZBTB7C	0.82	0.70	−14.73	1	1	−14.73
			SP8	0.80	0.68	−15.12	1	1	−15.12
			RUNX2	0.88	0.70	−20.51	2	0.5	−10.25
		T_H_1	HMBOX1	0.83	0.66	−20.33	2	0.5	−10.16
			TFCP2	0.83	0.73	−12.23	1	1	−12.23
			ARNT::HIF1A	0.80	0.63	−20.79	2	0.5	−10.39
*IL17C*	rs11646542 (C/A)	MELANO	RUNX2	0.89	0.71	−20.29	1	1	−20.29
		NHEK	RUNX2	0.89	0.71	−20.29	1	1	−20.29
*IL1*7*RA*	rs4819958 (C/T)	T_H_1	PAX1	0.81	0.73	−10.08	1	1	−10.08
	rs4819958 (G/A)	MONOCYTES-CD14^+ _^RO01746	NR2F1	0.80	0.66	−17.64	1	1	−17.64
			TFAP2C (var.3)	0.81	0.68	−15.50	1	1	−15.50
			TFAP2B (var.3)	0.81	0.69	−15.09	1	1	−15.09
*S100A7*	rs12049559 (C/T)	NHEK	ELF1	0.82	0.70	−13.67	1	1	−13.67
		HAEpiC	ELF1	0.82	0.70	−13.67	1	1	−13.67
*TNF*	rs361525 (G/A)	HAEpiC	MSX2	0.81	0.59	−27.16	2	0.5	−13.58
			MIXL1	0.83	0.66	−20.20	2	0.5	−10.10
			PRRX1	0.81	0.65	−19.72	1	1	−19.72
			PAX4	0.81	0.72	−11.77	1	1	−11.77
			GSC	0.82	0.72	−12.58	1	1	−12.58
			JUND	0.82	0.71	−13.22	1	1	−13.22
			HESX1	0.80	0.63	−21.06	1	1	−21.06
			SREBF2	0.81	0.69	−14.10	1	1	−14.10
	rs1800629 (A/G)	HAEpiC	JUN	0.81	0.70	−12.87	1	1	−12.87
			TCF4	0.81	0.73	−10.41	1	1	−10.41
			GSC	0.84	0.73	−12.39	1	1	−12.39
			MNT	0.84	0.75	−10.22	1	1	−10.22
			MAX	0.82	0.73	−10.64	1	1	−10.64

a Negative values in FIF indicate a prediction of decreased affinity between TF-DNA of minor allele compared with major allele. Positive values in FIF indicate a prediction of increased affinity between TF-DNA of minor allele compared with major allele. Positive values of FIF ≥10 suggest the creation of a new TFBS (unpublished results).

**Fig. 1:**
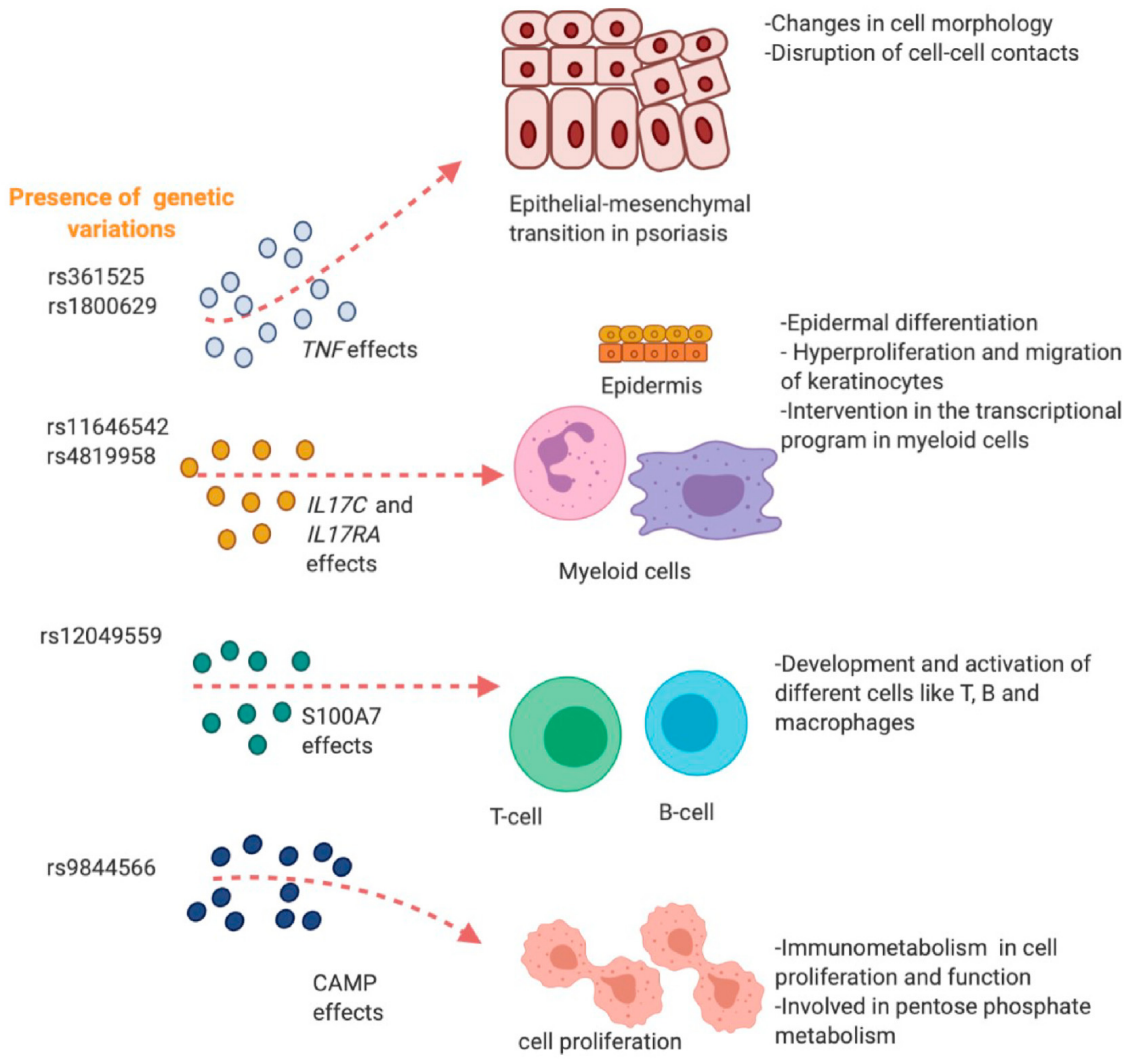
Proposed impact of the SNPClinic-predicted rSNPs in psoriasis (“ALL” population from 1000 Genomes Project). Figure was designed in Biorender by AVRR (https://biorender.com/)

**Table 3:** Data of eQTLs in four open databases. Confirmed true eQTLs (predicted by SNPClinic v.1.0) are shown in bold numbers.

GENE	rSNP	SNPClinic/ENCODE cell lines	GTEx	EBI-EMBL	ENSEMBL	ENCODE (Phase 3)
			(m-value^a^)	Expression Atlas (TPM/FPKM^c^)	Effect size^d^	Gene expression^f^
*CAMP*	rs9844566	NHEK	**1**	**2.6 (low)**	**−0.85**	**0.14**
		T_H_1	**0.96**	-2.4	**−0.55**	**0.01**
*IL17C*	rs11646542	MELANO	**1**	**4.8 (low)**	**−0.21**	NM^g^
		NHEK	**1**	-1.8	**−0.24**	NM
*IL1*7RA	rs4819958	T_H_1	**1**	-1.6	**−0.18**	**4.2**
		MONOCYTES-CD14^+^	**1**	**6.8(low)**	**−0.29**	**6.04**
*S100A7*	rs12049559	NHEK	NA^b^	**4.5(low)**	**0.14**	NM
		HAEpiC	NA	**2.2(low)**	**0.14**	NM
*TNF*	rs361525	HAEpiC	**1**	**2.2(low)**	NC^e^	NM
	rs1800629	HAEpiC	**1**	**1.2** (low)	NC	NM

a m-value m -value:<0.1 the tissue/cell line is predicted to not have an eQTL effect; >0.9: the tissue/cell line is predicted to have an eQTL effect.

b Not available (see Supplementary. Fig.1)

c TPM/FPKM ≤0.5 : expression level is below cutoff; TPM/FPKM from 0.5 to 10: expression level is low.

d Effect size: Effect of the alternative allele (ALT) relative to the reference allele (REF). The eQTL effect allele is the ALT allele.

e No gene or transcript consequences.

f Gene Expression Profiles by RNA-seq presented in log^2^ (TPM/FPKM+0.01).

g NM: No matching Candidate cis-Regulatory Elements (cCREs).

**Fig. 2:**
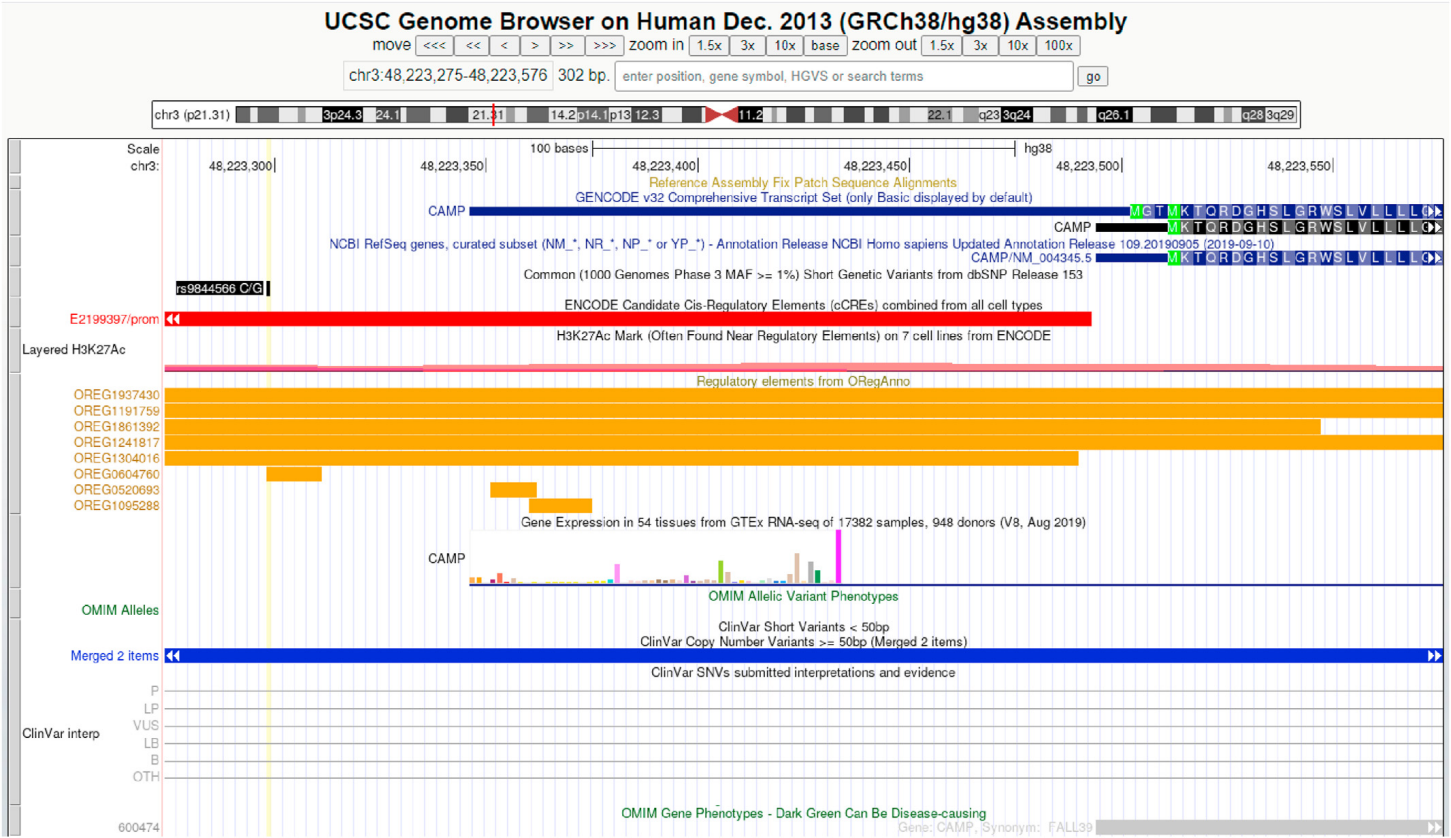
Genomic and epigenomic context (gene expression and histone markers as inputs of SNPClinic v.1.0) of rSNP rs9844566 (yellow vertical line) in proximal promoter of *CAMP* gene (cathelicidin LL-37). Figure generated at UCSC Genome Browser visual tool. (For interpretation of the references to colour in this figure legend, the reader is referred to the Web version of this article.)

**Fig. 3:**
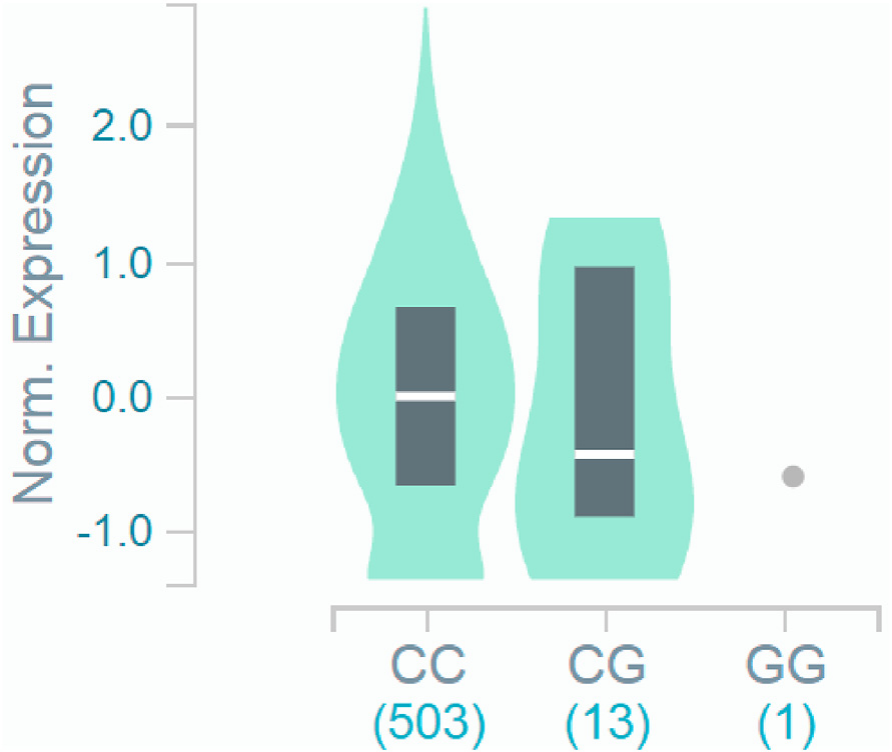
Violin plots of relative gene expression impacted by rs9844566 eQTL of *CAMP* gene (Chr.3) in not sun-exposed suprapubic skin (GTEx portal). White bar= median. Numbers in *x*-axis indicate number of individuals.

**Fig. 4:**
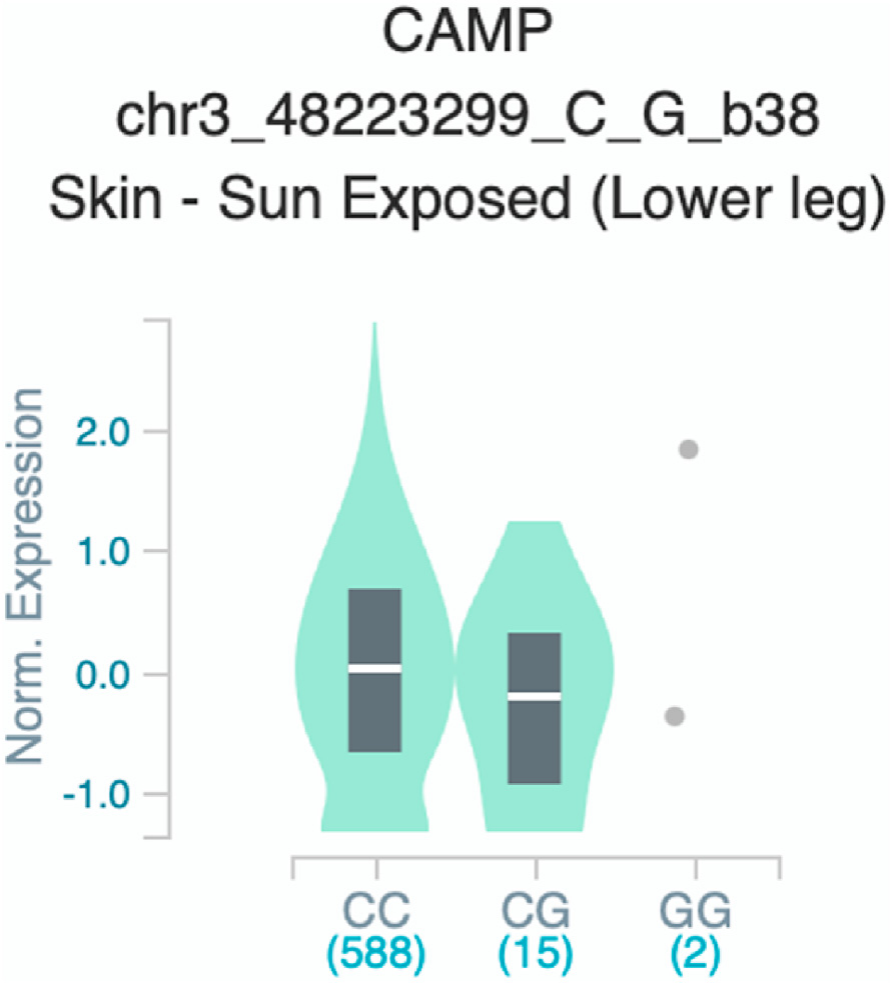
Violin plots of relative gene expression impacted by rs9844566 eQTL of *CAMP* gene (Chr.3) in Skin - Sun Exposed [Lower leg] (GTEx portal). White bar= median. Numbers in *x*-axis indicate number of individuals.

**Fig. 5:**
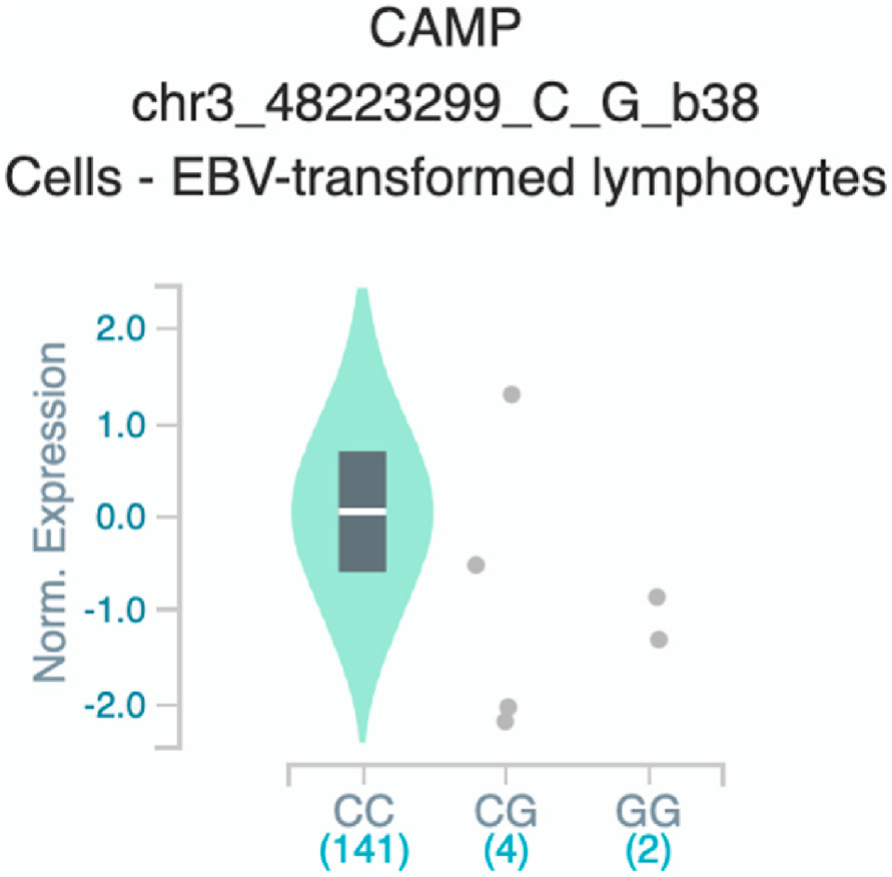
Violin plots of relative gene expression impacted by rs9844566 eQTL of *CAMP* gene (Chr.3) in Cells_EBV-transformed_lymphocytes (GTEx portal). White bar= median. Numbers in *x*-axis indicate number of individuals.

**Fig. 6:**
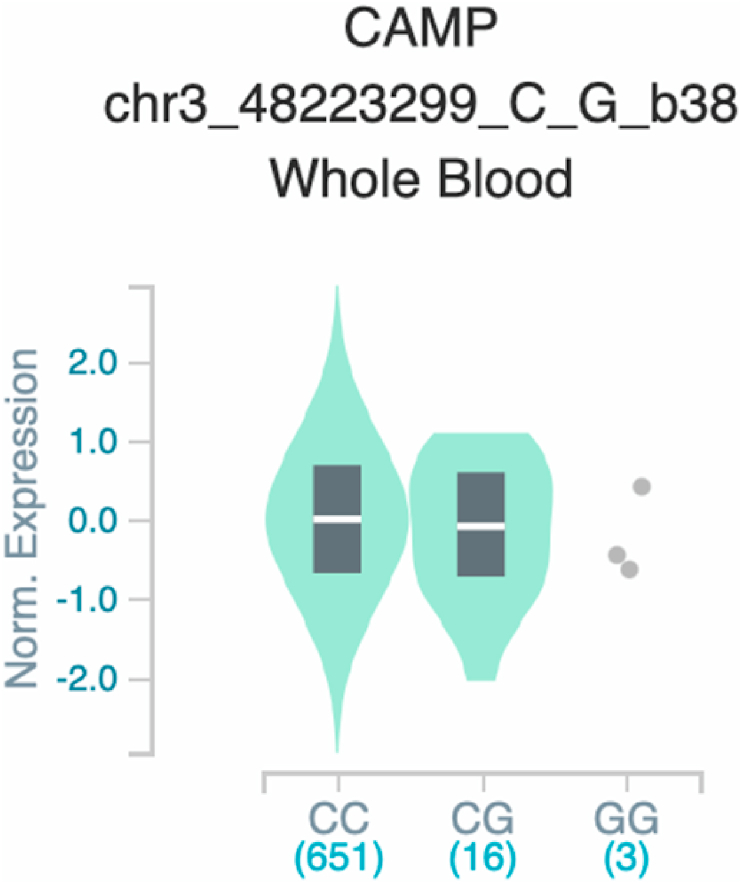
Violin plots of relative gene expression impacted by rs9844566 eQTL of *CAMP* gene (Chr.3) in Whole_Blood (GTEx portal). White bar= median. Numbers in *x*-axis indicate number of individuals.

**Fig. 7:**
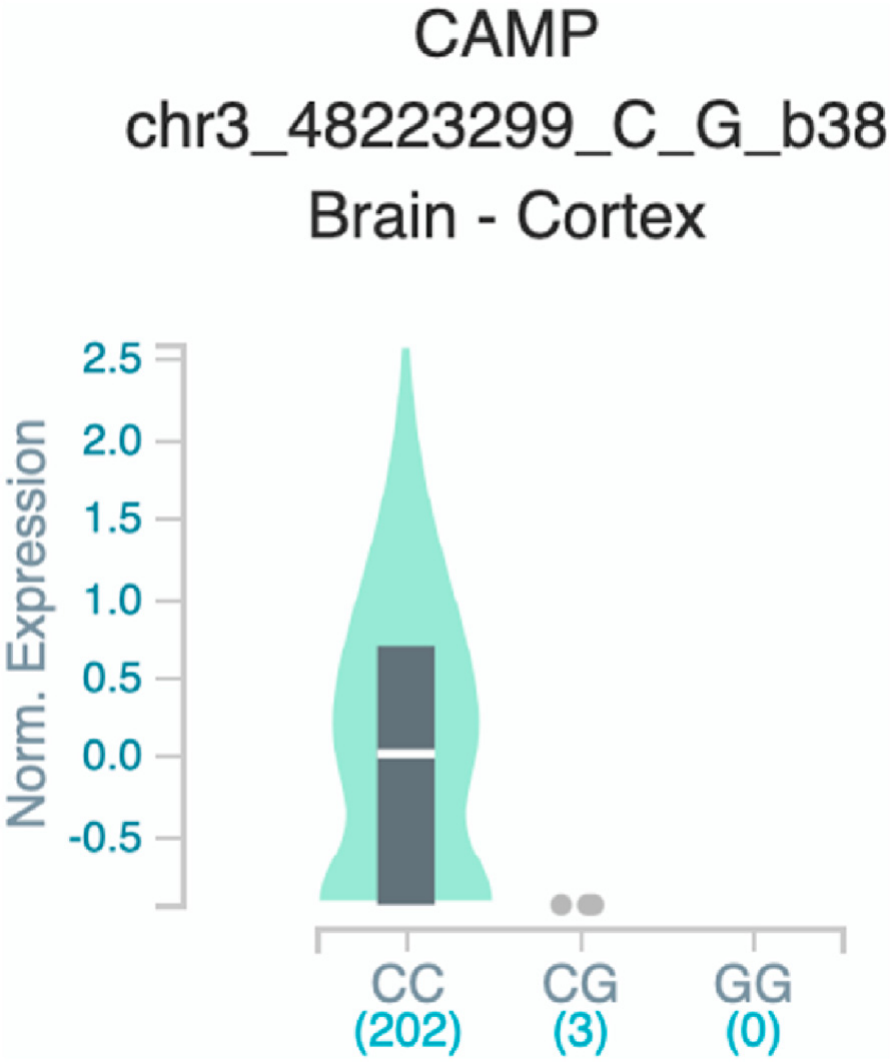
Violin plots of relative gene expression impacted by rs9844566 eQTL of *CAMP* gene (Chr.3) in Brain_Cortex (GTEx portal). White bar= median. Numbers in *x*-axis indicate number of individuals.

**Fig. 8:**
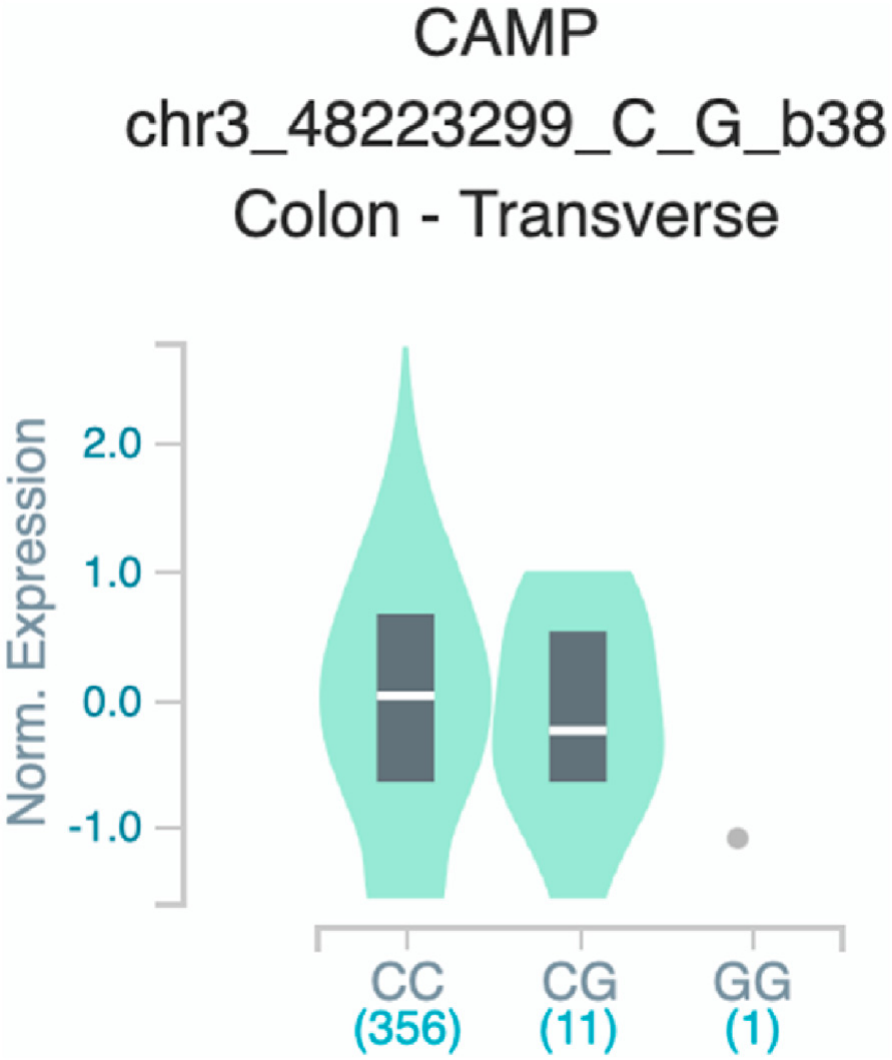
Violin plots of relative gene expression impacted by rs9844566 eQTL of *CAMP* gene (Chr.3) in Colon_Transverse (GTEx portal). White bar= median. Numbers in *x*-axis indicate number of individuals.

**Fig. 9:**
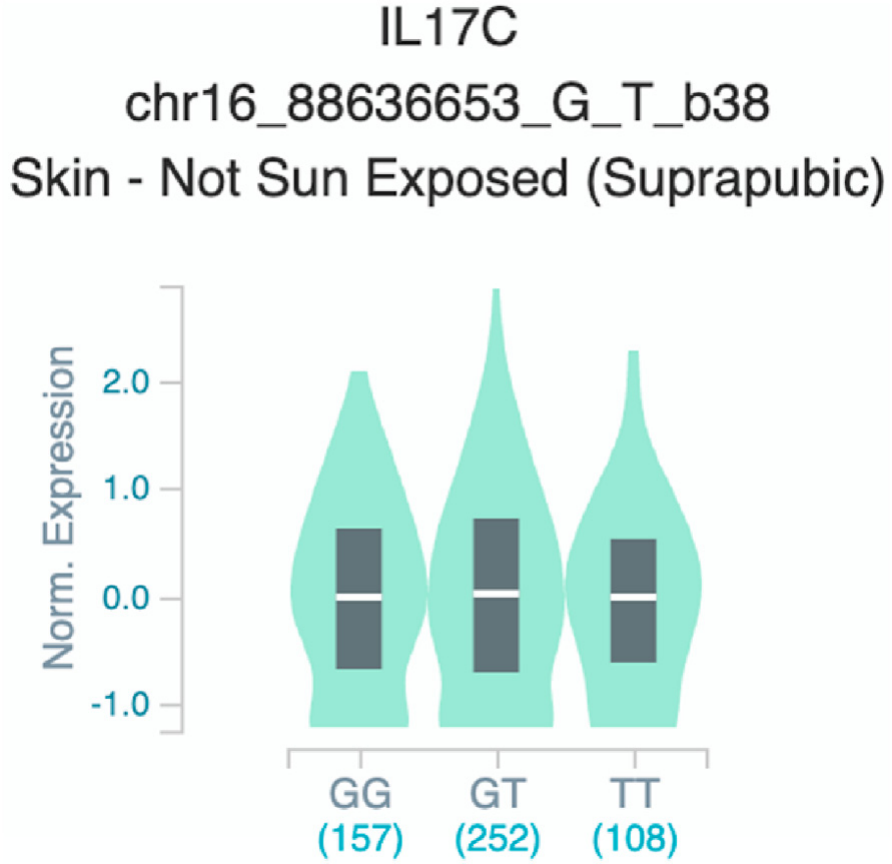
Violin plots of relative gene expression impacted by rs11646542 eQTL of *IL17C* gene (Chr.16) in Not_Sun_Exposed_Suprapubic skin (GTEx portal). White bar= median. Numbers in *x*-axis indicate number of individuals.

**Fig. 10:**
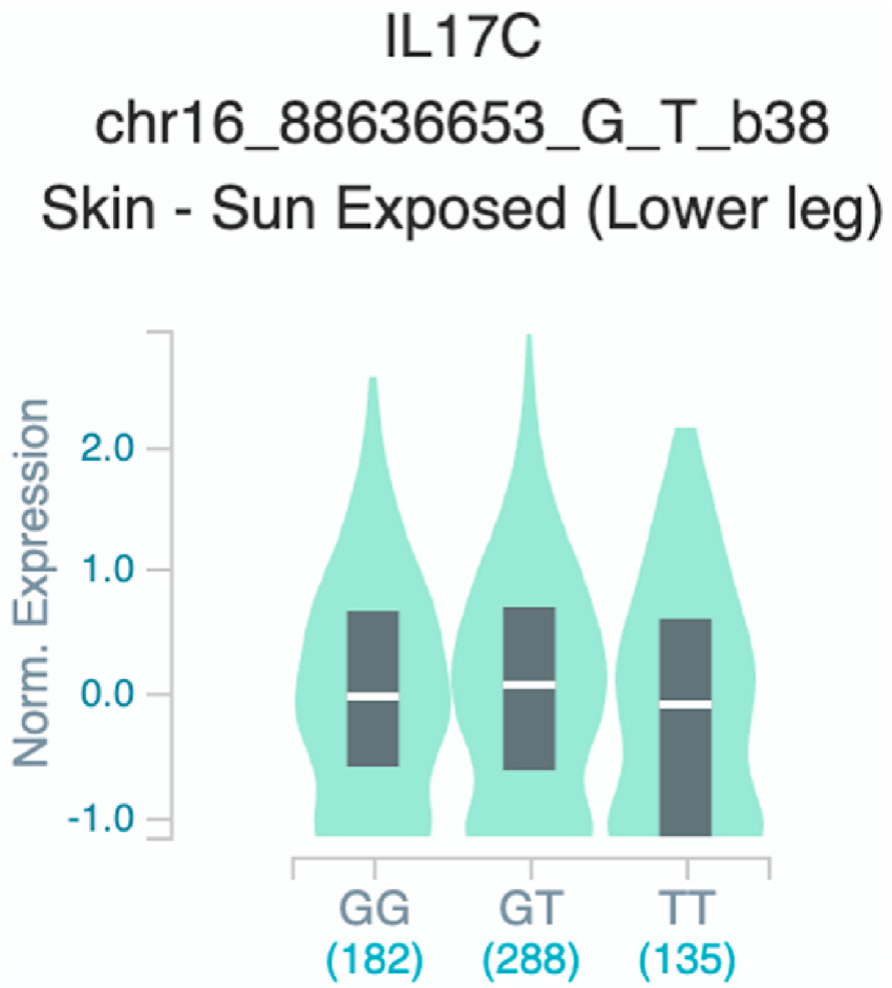
Violin plots of relative gene expression impacted by rs11646542 eQTL of *IL17C* gene (Chr.16) in Sun-Exposed Lower leg skin (GTEx portal). White bar= median. Numbers in *x*-axis indicate number of individuals.

**Fig. 11:**
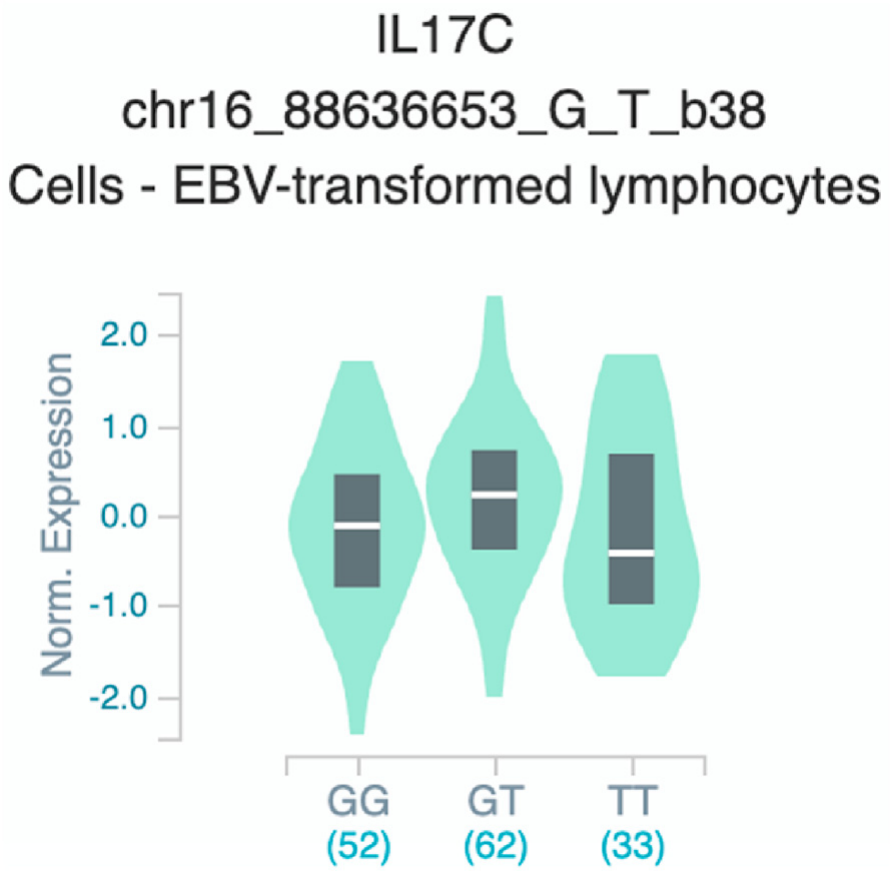
Violin plots of relative gene expression impacted by rs11646542 eQTL of *IL17C* gene (Chr.16) in Cells_EBV-transformed_lymphocytes (GTEx portal). White bar= median. Numbers in *x*-axis indicate number of individuals.

**Fig. 12:**
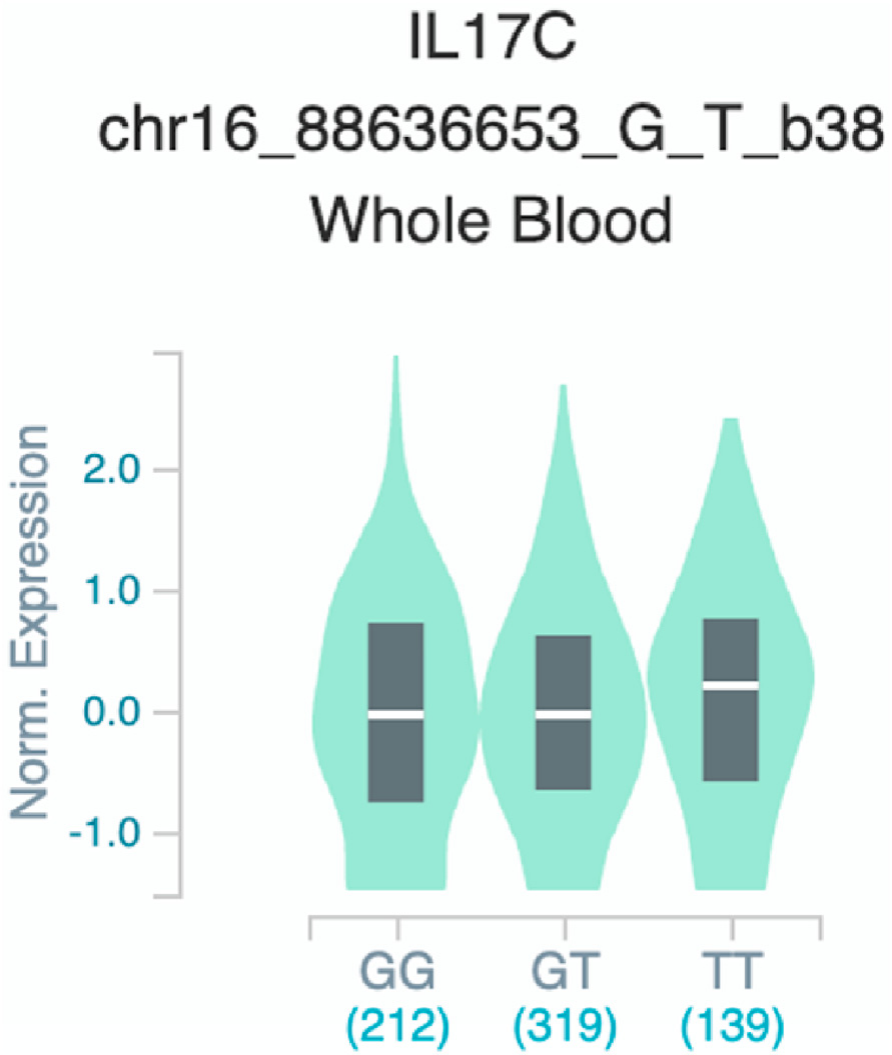
Violin plots of relative gene expression impacted by rs11646542 eQTL of *IL17C* gene (Chr.16) in Whole_Blood (GTEx portal). White bar= median. Numbers in *x*-axis indicate number of individuals.

**Fig. 13:**
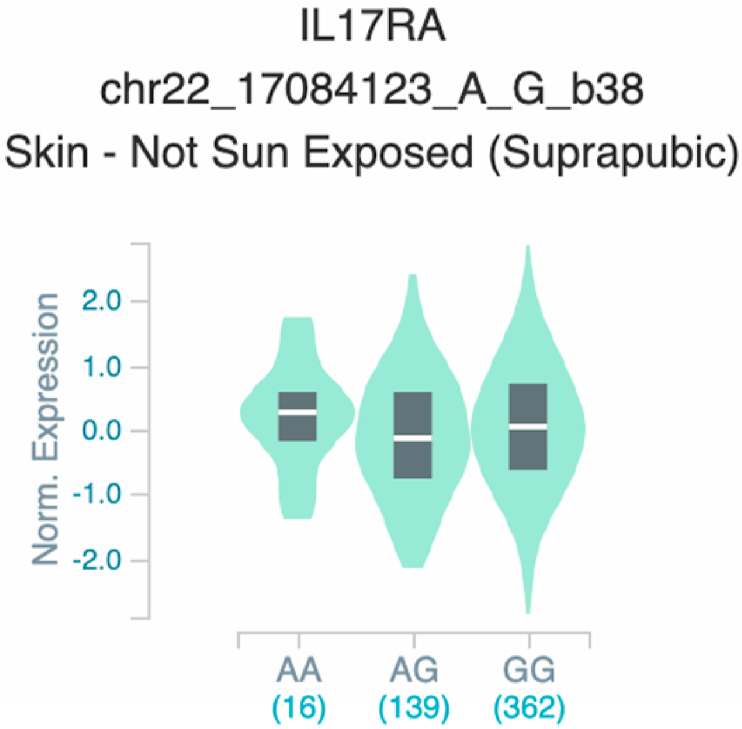
Violin plots of relative gene expression impacted by rs4819958 eQTL of *IL1*7*RA* gene (Chr.22) in Not Sun-Exposed Suprapubic skin (GTEx portal). White bar= median. Numbers in *x*-axis indicate number of individuals.

**Fig. 14:**
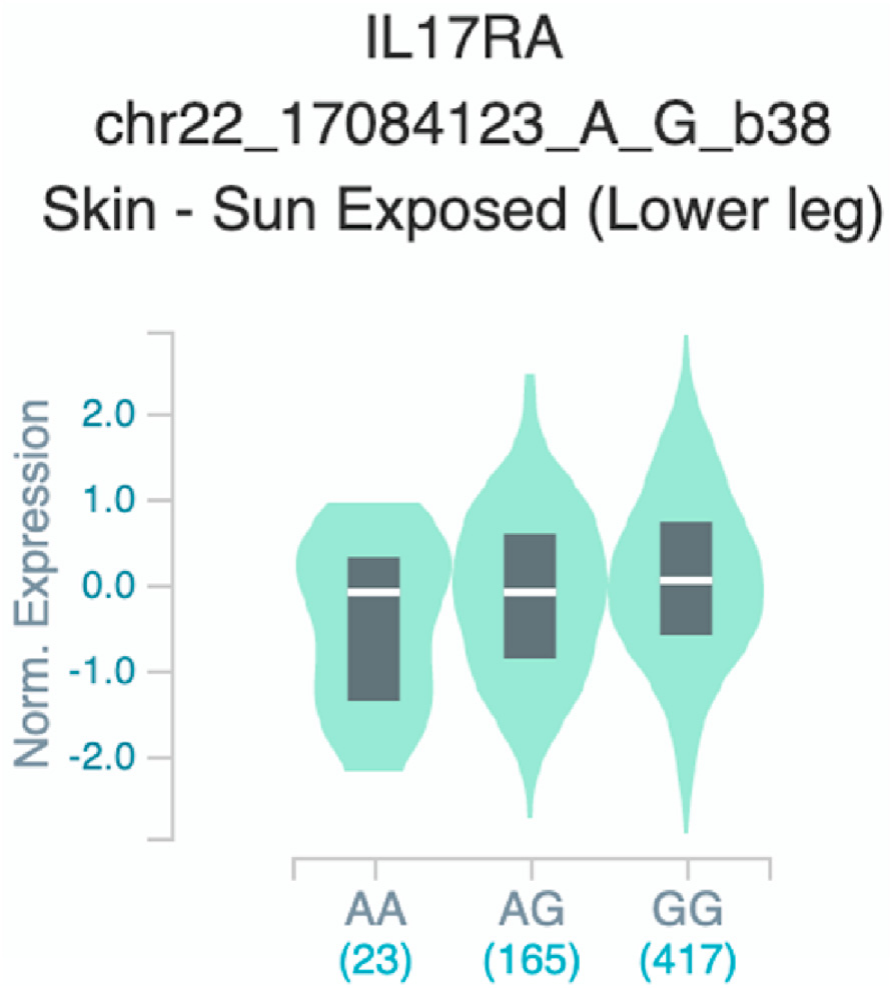
Violin plots of relative gene expression impacted by rs4819958 eQTL of *IL1*7*RA* gene (Chr.22) in Sun_-Exposed Lower leg skin (GTEx portal). White bar= median. Numbers in *x*-axis indicate number of individuals.

**Fig. 15:**
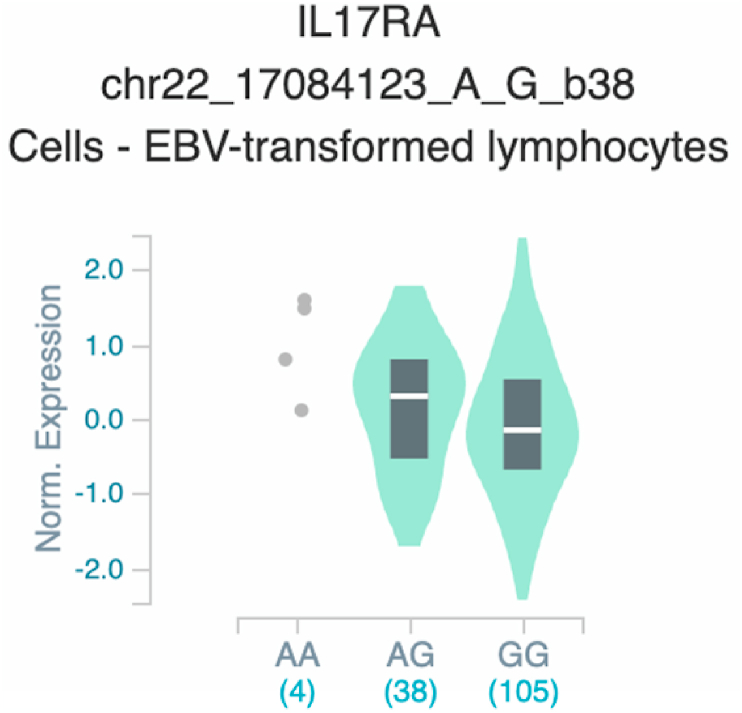
Violin plots of relative gene expression impacted by rs4819958 eQTL of *IL1*7*RA* gene (Chr.22) in Cells_EBV-transformed_lymphocytes (GTEx portal). White bar= median. Numbers in *x*-axis indicate number of individuals.

**Fig. 16:**
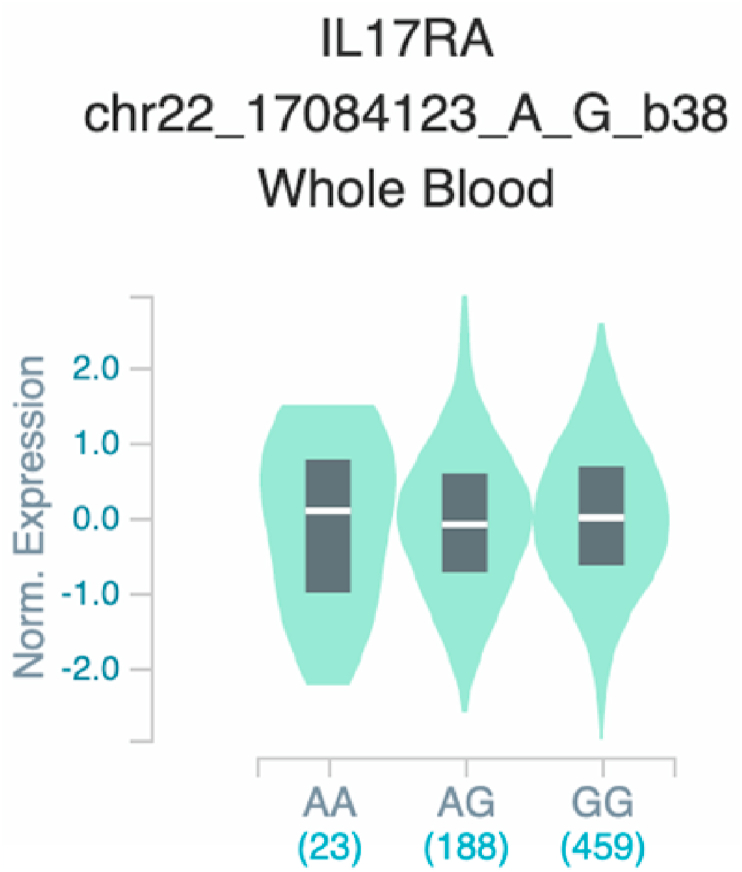
Violin plots of relative gene expression impacted by rs4819958 eQTL of *IL1*7*RA* gene (Chr.22) in Whole Blood (GTEx portal). White bar= median. Numbers in *x*-axis indicate number of individuals.

**Fig. 17:**
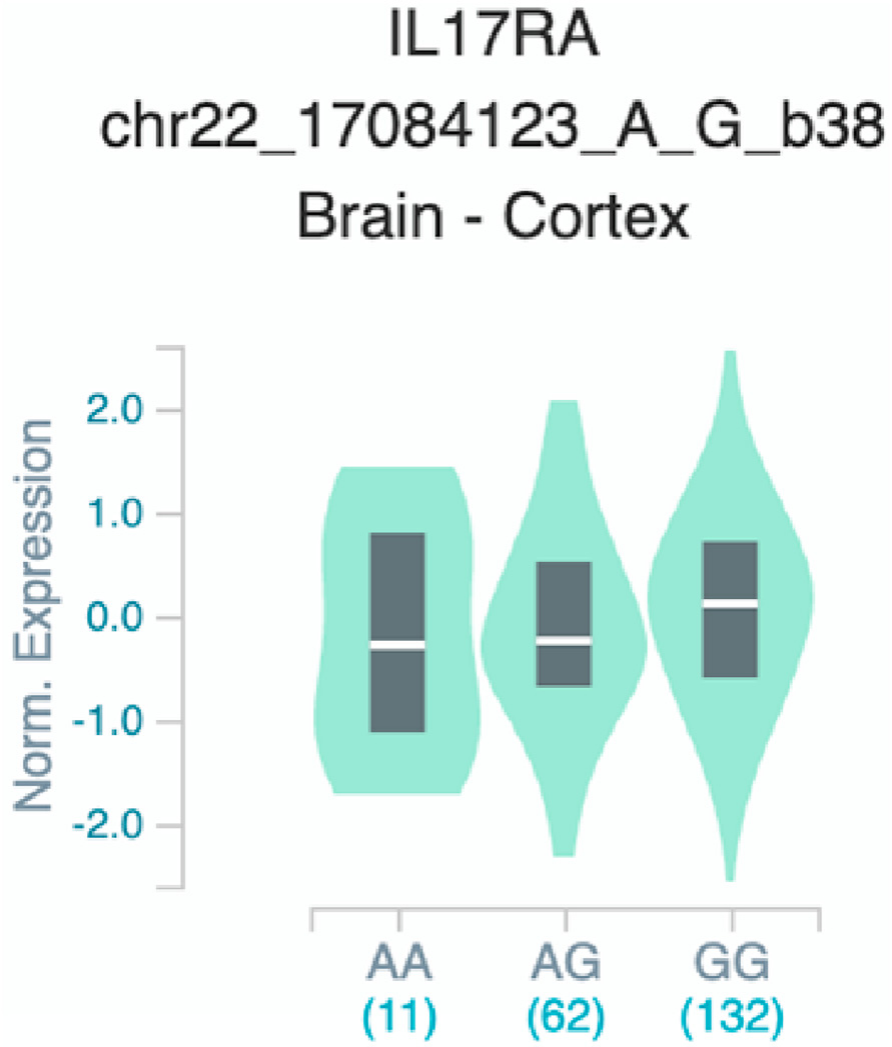
Violin plots of relative gene expression impacted by rs4819958 eQTL of *IL1*7*RA* gene (Chr.22) in Brain_Cortex (GTEx portal). White bar= median. Numbers in *x*-axis indicate number of individuals.

**Fig. 18:**
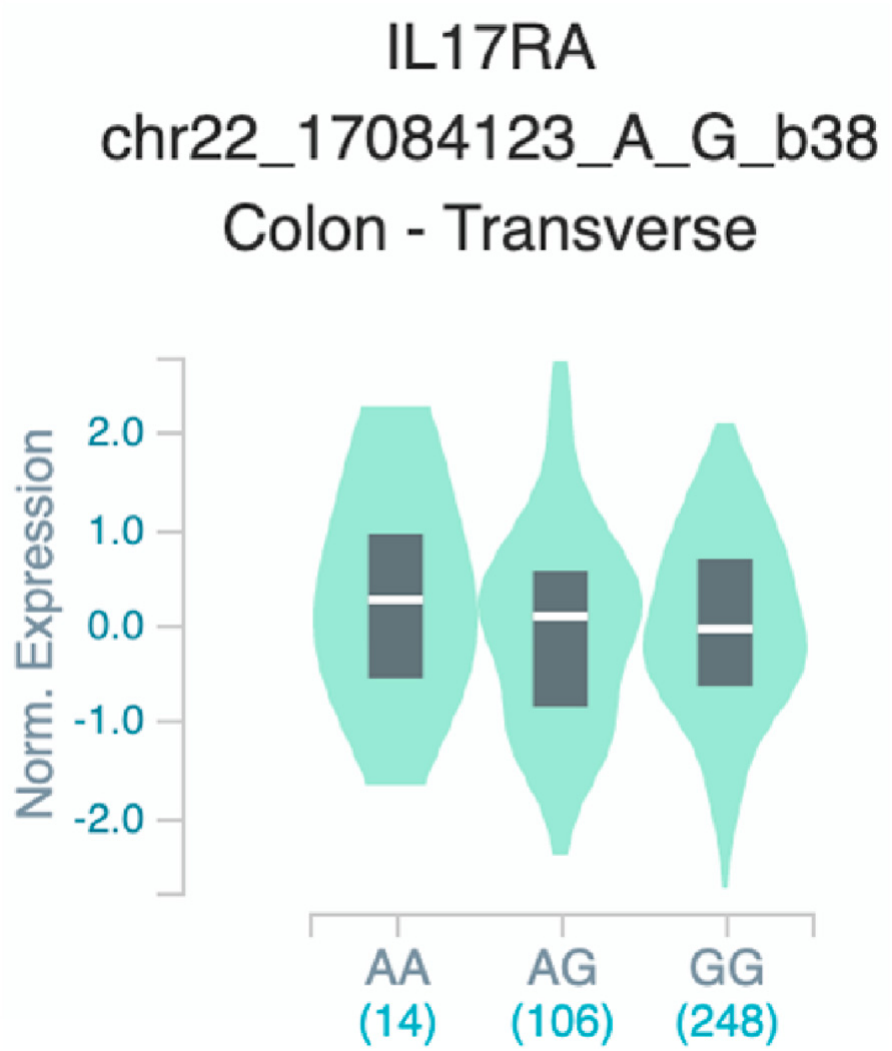
Violin plots of relative gene expression impacted by rs4819958 eQTL of *IL1*7*RA* gene (Chr.22) in Colon_Transverse (GTEx portal). White bar= median. Numbers in *x*-axis indicate number of individuals.

**Fig. 19:**
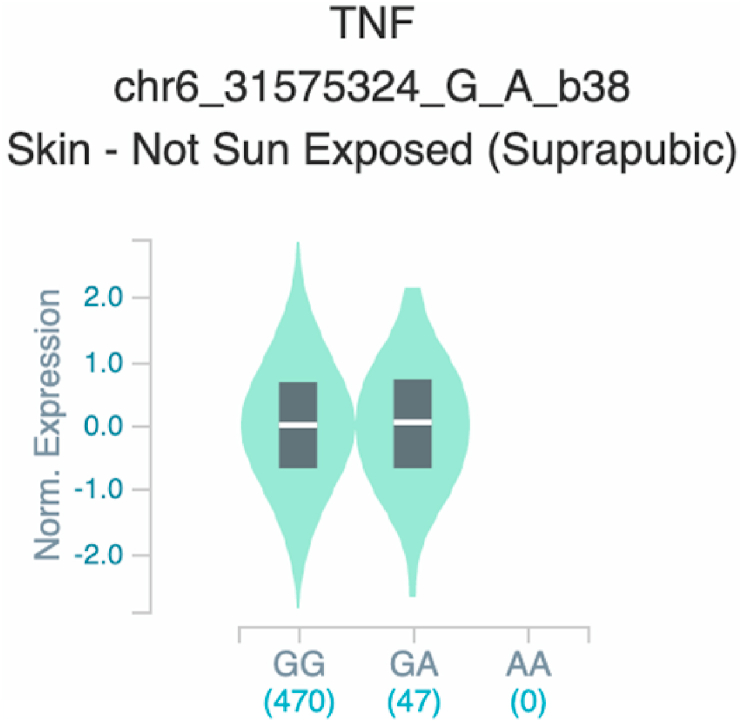
Violin plots of relative gene expression impacted by rs361525 eQTL of *TNF* gene (Chr.6) in Not Sun-Exposed Suprapubic skin (GTEx portal). White bar= median. Numbers in *x*-axis indicate number of individuals.

**Fig. 20:**
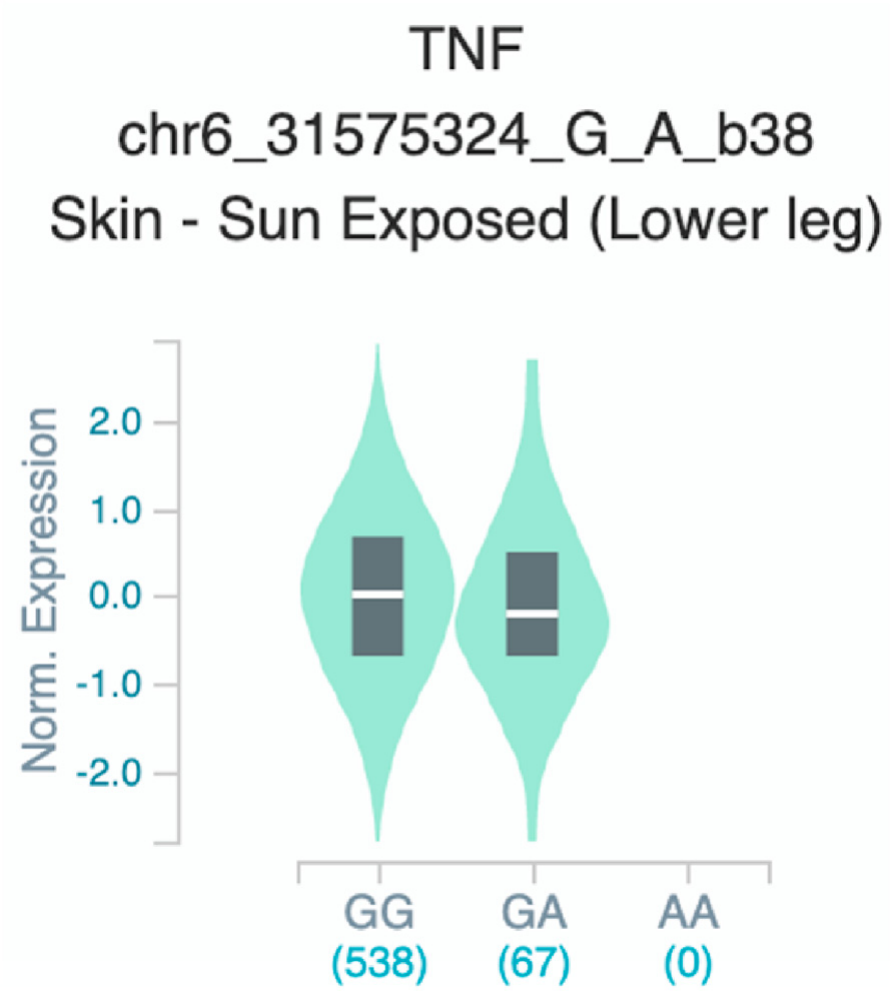
Violin plots of relative gene expression impacted by rs361525 eQTL of *TNF* gene (Chr.6) in Sun-Exposed Lower leg skin (GTEx portal). White bar= median. Numbers in *x*-axis indicate number of individuals.

**Fig. 21:**
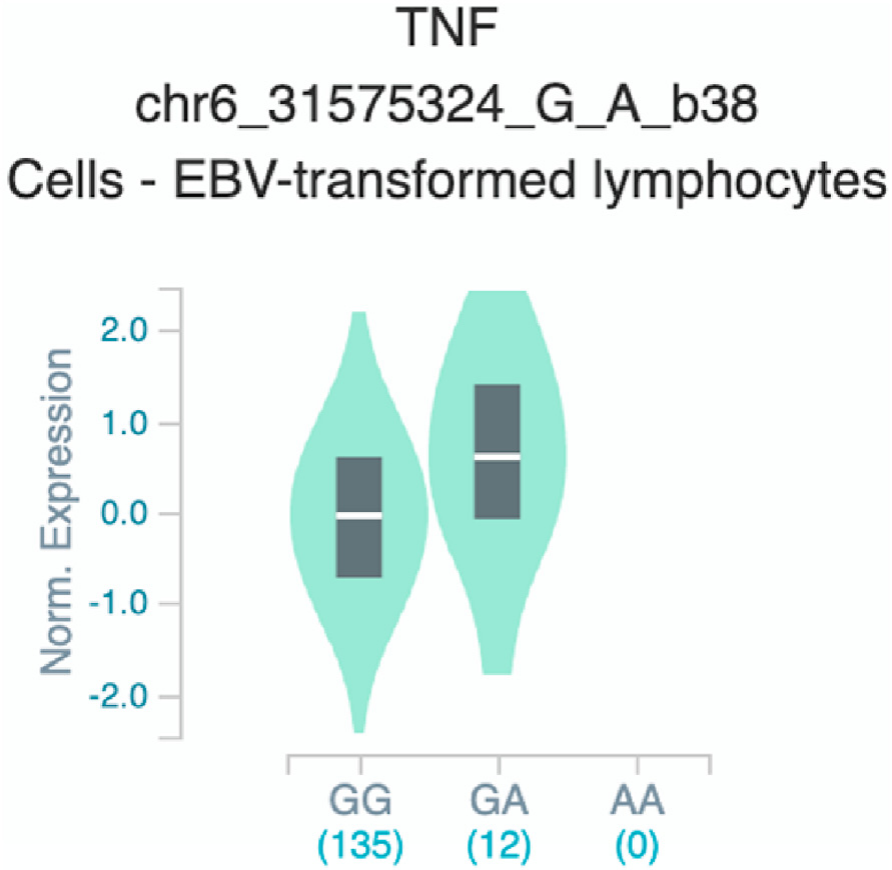
Violin plots of relative gene expression impacted by rs361525 eQTL of *TNF* gene (Chr.6) in Cells_EBV-transformed_lymphocytes (GTEx portal). White bar= median. Numbers in *x*-axis indicate number of individuals.

**Fig. 22:**
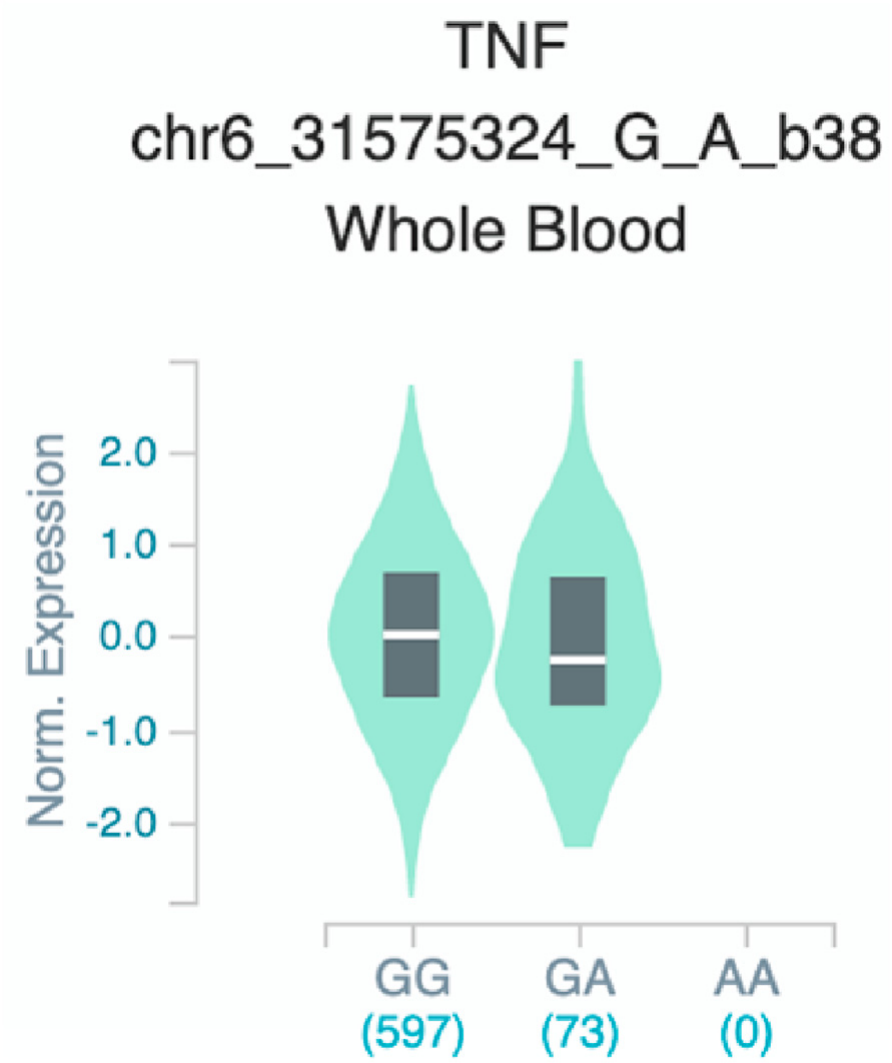
Violin plots of relative gene expression impacted by rs361525 eQTL of *TNF* gene (Chr.6) in Whole_Blood (GTEx portal). White bar= median. Numbers in *x*-axis indicate number of individuals.

**Fig. 23:**
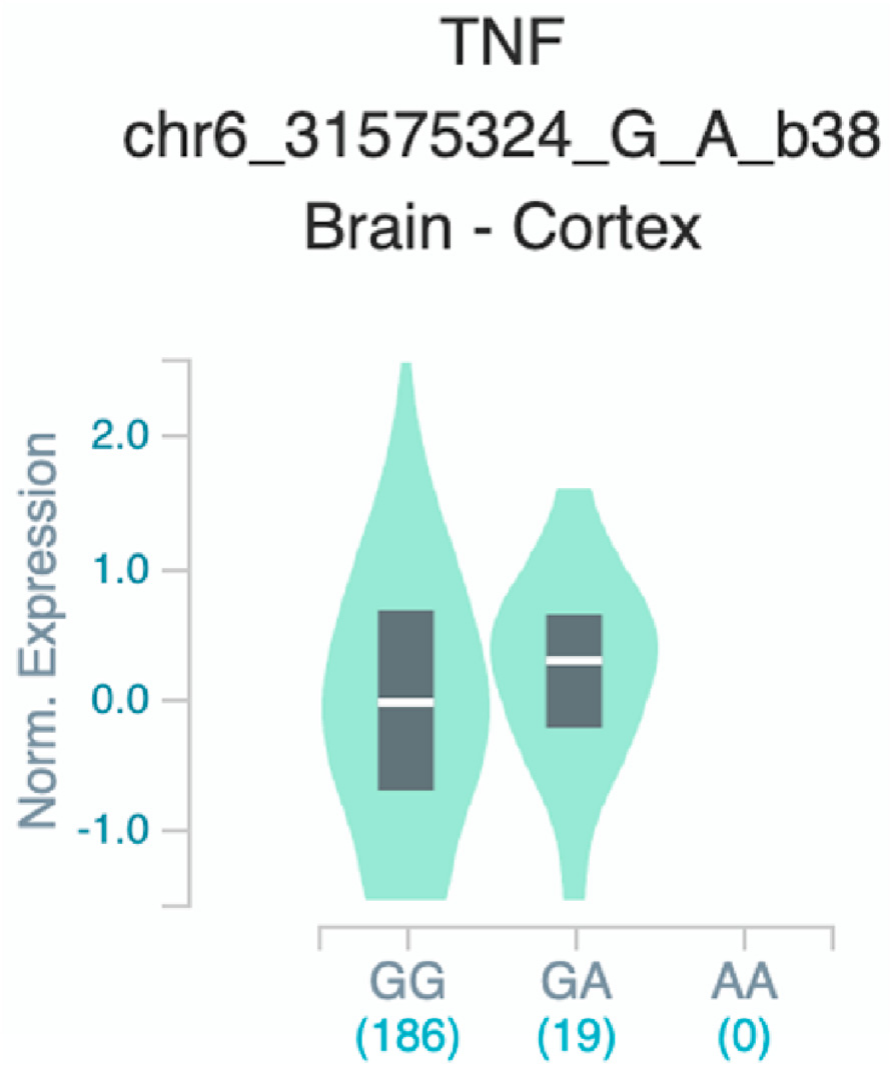
Violin plots of relative gene expression impacted by rs361525 eQTL of *TNF* gene (Chr.6) in Brain_Cortex (GTEx portal). White bar= median. Numbers in *x*-axis indicate number of individuals.

**Fig. 24:**
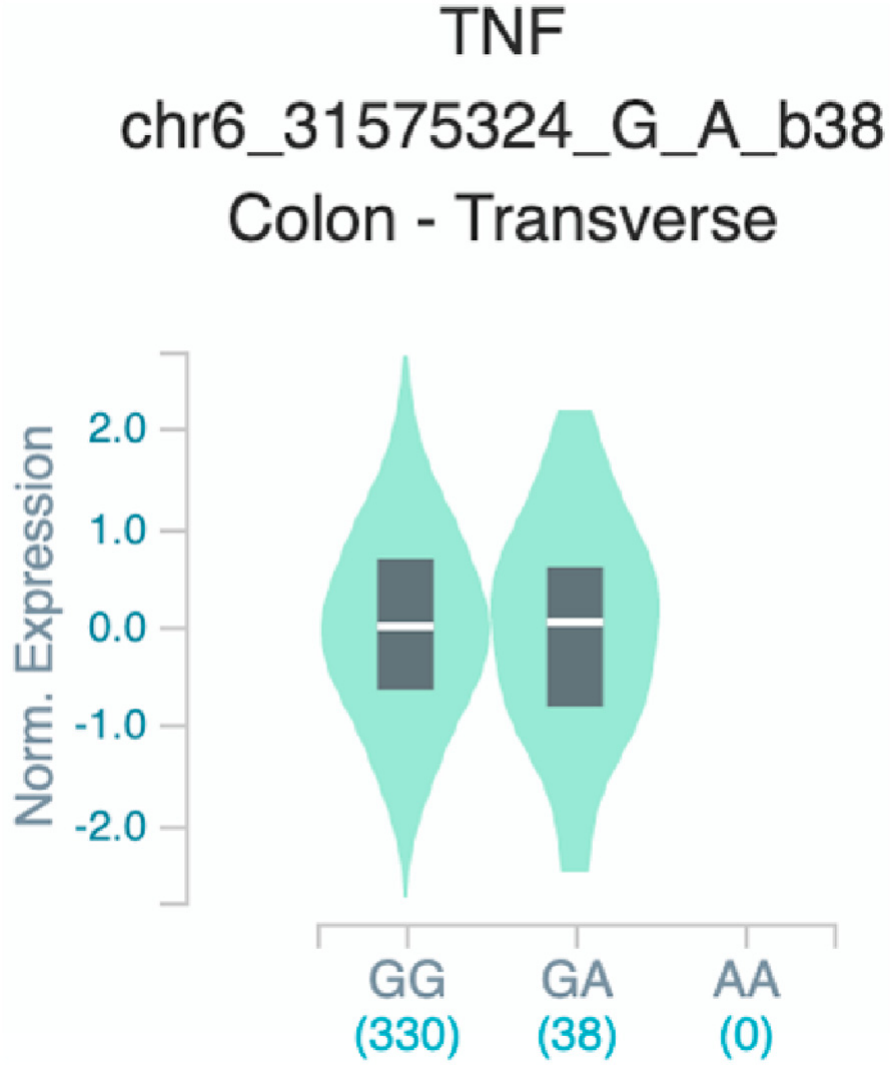
Violin plots of relative gene expression impacted by rs361525 eQTL of *TNF* gene (Chr.6) in Colon-Transverse (GTEx portal). White bar= median. Numbers in *x*-axis indicate number of individuals.

**Fig. 25:**
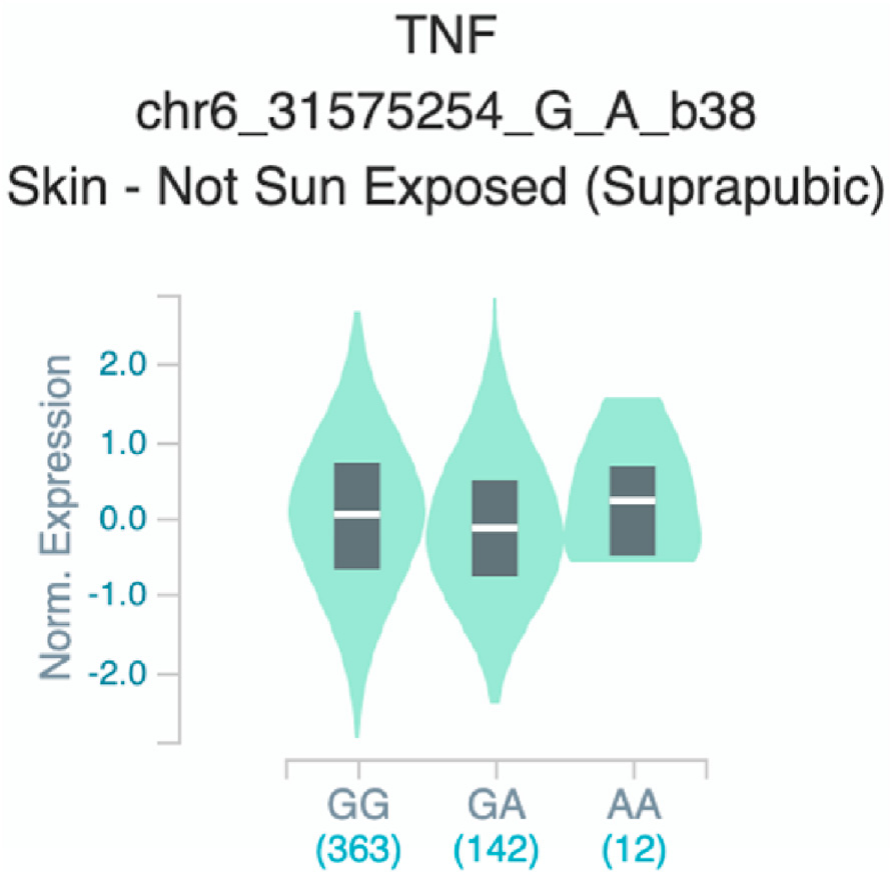
Violin plots of relative gene expression impacted by rs1800629 eQTL of *TNF* gene (Chr.6) in Not Sun-Exposed Suprapubic skin (GTEx portal). White bar= median. Numbers in *x*-axis indicate number of individuals.

**Fig. 26:**
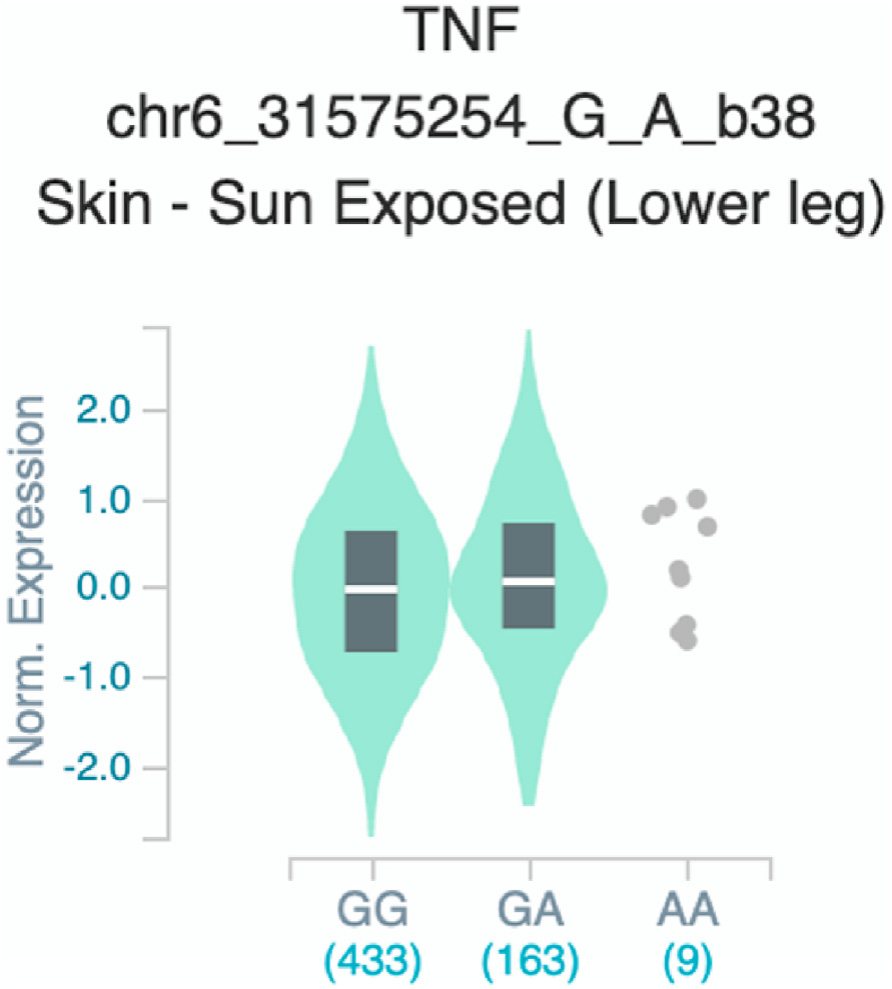
Violin plots of relative gene expression impacted by rs1800629 eQTL of *TNF* gene (Chr.6) inSun-Exposed Lowerleg skin (GTEx portal). White bar= median. Numbers in *x*-axis indicate number of individuals.

**Fig. 27:**
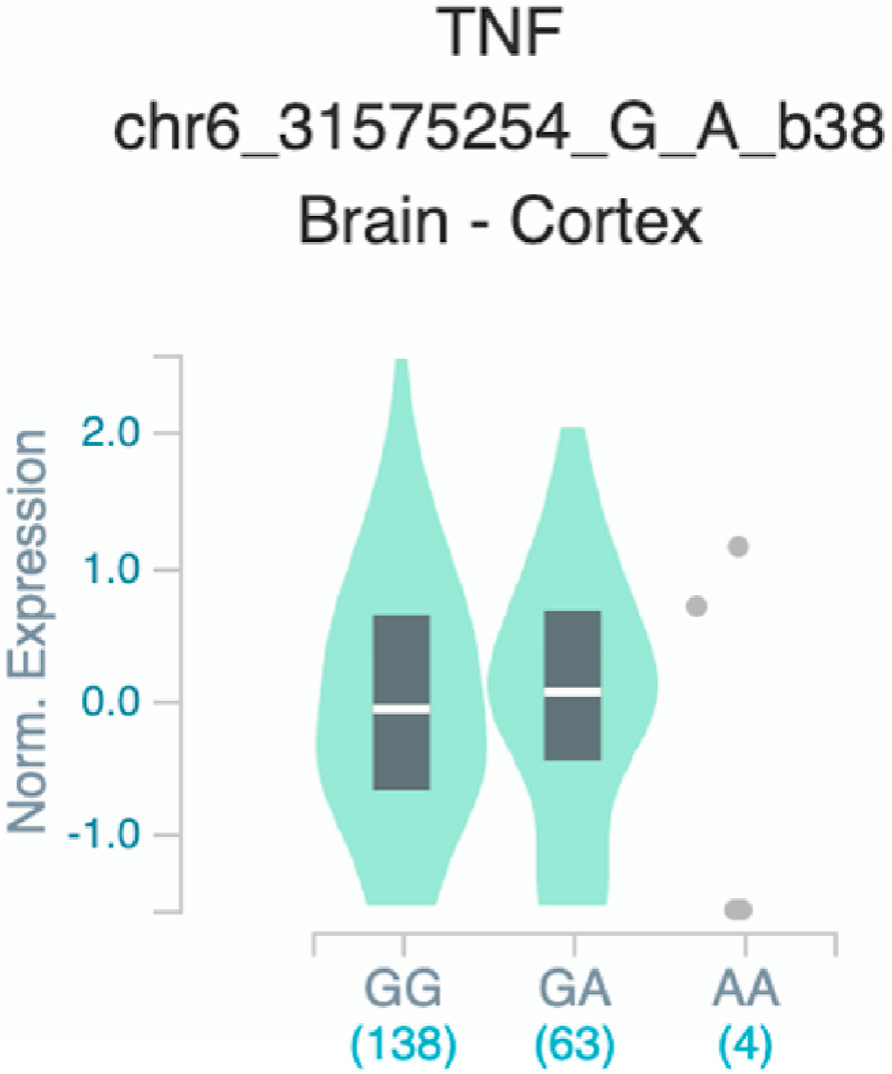
Violin plots of relative gene expression impacted by rs1800629 eQTL of TNF gene (Chr.6) in brain cortex (GTEx portal). White bar= median. Numbers in *x*-axis indicate number of individuals.

**Fig. 28:**
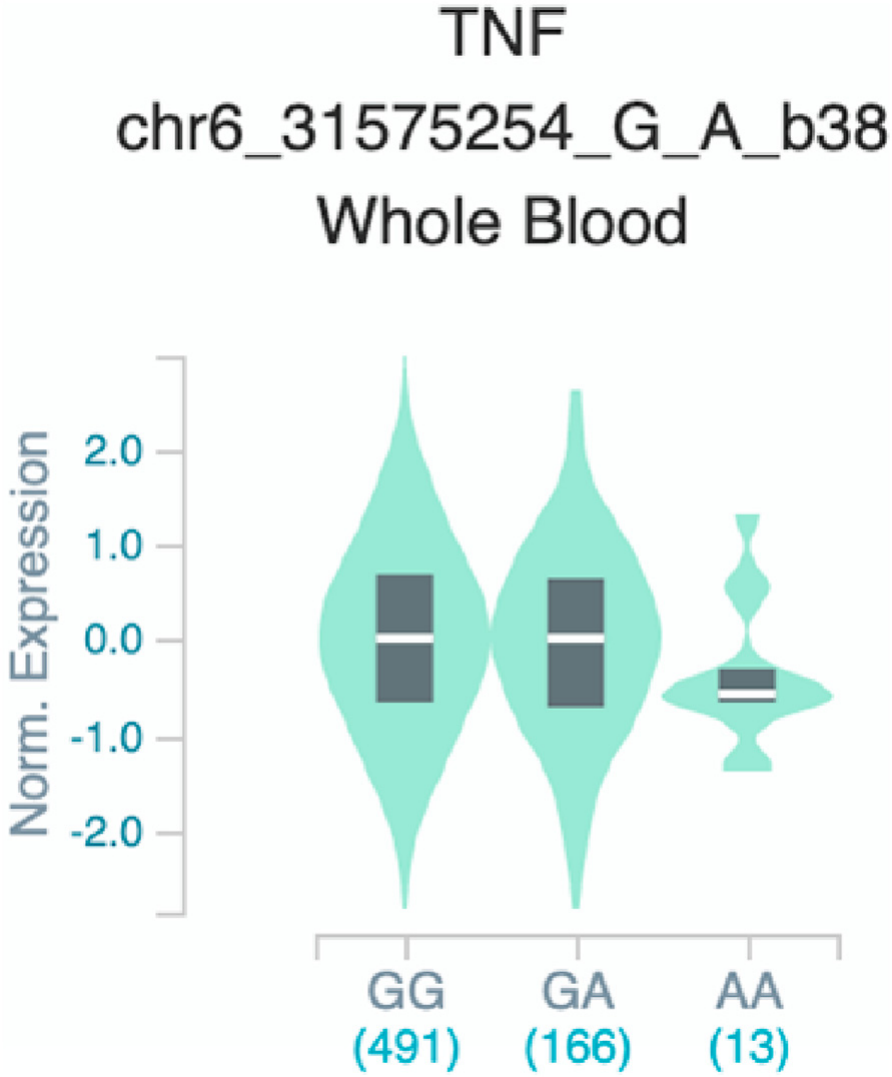
Violin plots of relative gene expression impacted by rs1800629 eQTL of *TNF* gene (Chr.6) in Whole_Blood (GTEx portal). White bar= median. Numbers in *x*-axis indicate number of individuals.

**Fig. 29:**
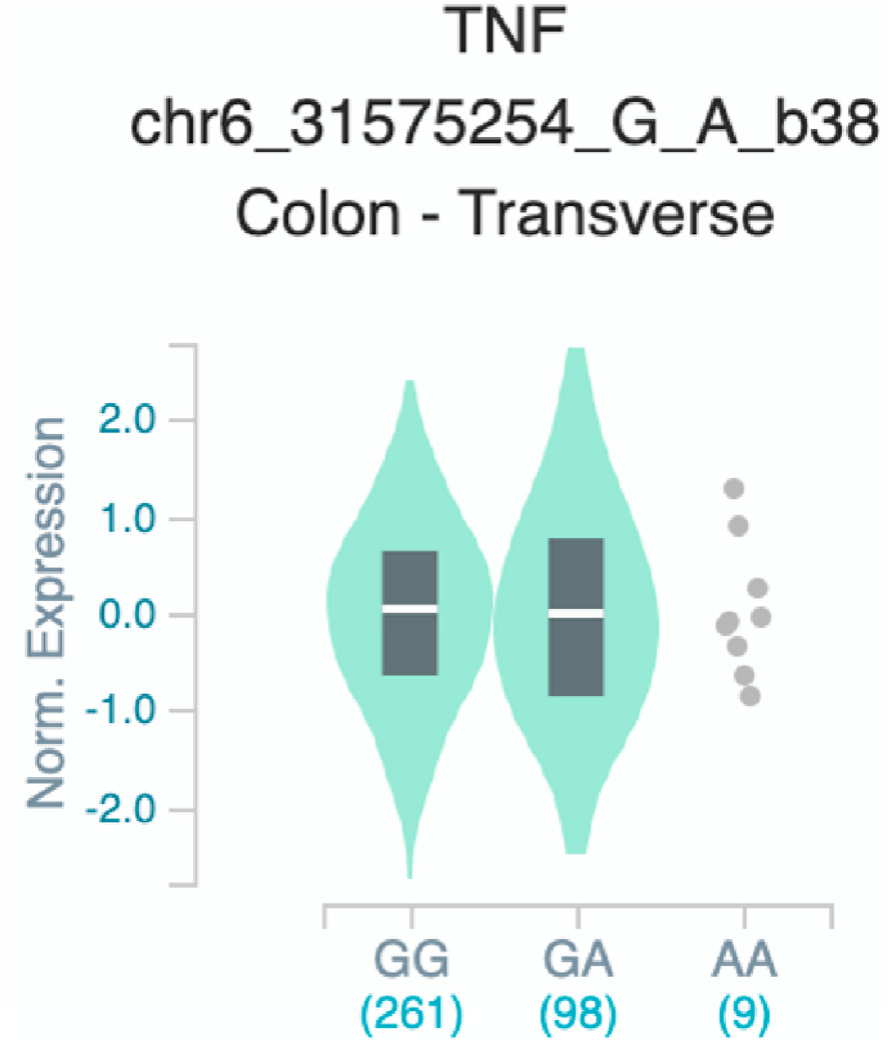
Violin plots of relative gene expression impacted by rs1800629 eQTL of *TNF* gene (Chr.6) in Colon_Transverse (GTEx portal). White bar= median. Numbers in *x*-axis indicate number of individuals.

It has been found three families of innate antimicrobial peptides and proteins: Cathelicidin (LL-37), S100 proteins and defensins that have been identified as potentially important players in psoriasis. These AMPs can be found in higher concentrations in psoriatic skin lesions, mainly in keratinocytes, phagocytes, T-cells, NK-cells, monocytes and mast cells [6].We have included *CAMP* (cathelicidin LL-37), *DEFB1* and *DEFB4* (defensins), *S100A7A,* *S100A7* (psoriasin) as well as *S100A8* and *S100A9* (which protein heterodimers form calprotectin) in this study. From these, we found rSNP in *CAMP and* *S100A7* genes. In the promoter region of *CAMP* gene in T_H_1 cell line, TFBS were altered for transcription factors HMBOX1, TFCP2 and ARNT:HIF1A when SNP rs9844566 is present. The same SNP is regulatory in NHEK altering TFBS for forkhead box P2 (FOXP2), SP8 and ZBTB7C, among others. Of these TFs just the HIF1A and FOXP2 were previously suggested by our group and others [15,19,20] as regulators of *CAMP* gene expression. SNP rs9844566 also was suggested as regulatory in A549, HL60 and CD14 ​cell lines [15]. FOXP2 is involved in embryonic development, immune disorders and cancer progression [21]. FOXP2 regulated p21 independently of both p53 and IL-6. *In vitro* evidence suggest that FOXP2 is involved in pentose phosphate metabolism by binding to ribose 5-phosphate isomerase A (RPIA) [48,50]. It is known that immunometabolism determines cell proliferation and function, as well as the homeostasis of the different lineages [22].

In psoriatic lesions, gene expression of *CAMP* (cathelicidin antimicrobial peptide, which encodes a precursor protein LL-37) is uncontrolled [23]. LL-37 is known to have the ability to increase the inflammatory response and facilitates the binding of nucleic acids to scavenger receptors leading to an autoinflammatory response. Thus LL-37 breaks tolerance to self-nucleic acids triggering inflammation in psoriasis [24]. *CAMP* gene, has binding sites for hypoxia-inducible factor 1 (HIF-1α) in the promoter region [19,20]. HIF-1α is considered as an immune modulator upregulated in hypoxia conditions such as systemic diseases, causing an increase in phagocytosis [25]. Besides its well-known initial role in psoriasis in breaking tolerance to self-RNA/DNA, our results suggest that cathelicidin could also participate in later events as hyperproliferation and differentiation of cells maybe in an oxygen-dependent manner. It is worth to mention that SNP rs9844566 has high frequency in African superpopulation (14.7%), frequent in American superpopulation (2%), rare in European (0.1%) and absent in Asian population [49].

IL-17 is a family of cytokines consisting of six members, with both amino acid sequence homology and highly conserved cysteine residues [26]. One of the main actions of IL-17 is the recruitment of neutrophils perpetuating local inflammation; this cytokine can synergize with highly proinflammatory cytokines such as TNF-α and IFN-γ [27]. The *IL17* gene family plays an important role against certain pathogens through the stimulation of the release of antimicrobial peptides, pro-inflammatory cytokines and chemokines [12]. IL17C is the most abundant IL-17 isoform in lesional skin in psoriasis, it can be found in keratinocytes, endothelial cells and leukocytes and share IL17RA as a co-receptor with IL17A*.* IL17C isoform increases the production of itself and of hBD-2 (*DEFB4 gene)*, S100A7/A8/A9 and IL-19 among other proteins enabling a pro-inflammatory positive feedback loop [28].

It was previously thought that the inflammatory profile of psoriasis was T_H_1, but more recently has been proved to be a clearly defined T_H_17 profile [27]. T_H_17 cells are differentiated from Naive CD4^+^ T cells that were stimulated by cytokines such as TGF-β, IL-6, IL-23 and have the ability to express IL-17, IL-21, IL-22, TNF- α, IL-6 and GM-CSF [29]. In *IL17C* gene, we found one putative rSNP in melanocytes and keratinocytes, with alteration in both cell lines of RUNX2 binding site, which is part of a set of “hub” transcription factors, whose motifs are enriched in many super-enhancers involved in epidermal differentiation [30]. These results are in agreement with hyperproliferation of keratinocytes, a hallmark of psoriasis.

The interleukin 17 receptor A gene (*IL1*7*RA*), is a single-pass transmembrane receptor expressed in all tissues examined to date. When IL17RA is in contact with its ligand, leads to an activation of the NF-κB pathway, inducing secretion of other proinflammatory cytokines [31] such as IL-1, IL-6, IL-8, TNF, MMPS. This secretion maintains inflammation and an increased cellular infiltrate [32]. In *IL1*7*RA*, eight SNPs were evaluated, but we found only rs4819958 as putative regulatory in the T_H_1 and monocyte cell line, with altered TFBS for paired box gene 1 (PAX1) and NR2F1. The ortholog gene in mice, *Pax1*, together with *Pax9*, are involved in organogenesis of thymus [33,34]. However, in humans its function has been scarcely studied. PAX1 is an interesting finding in psoriasis-related genes, due to the pivotal role of thymus in self-recognition. Regarding TFAP2C and TFAP2B, these are the most important repressors of the transcriptional program in myeloid cells [35]. In particular, TFAP2C is associated with hyperproliferation and migration of keratinocytes in psoriasis [36]. In psoriasis, there is a downregulation of *TFAP2C* and *TFAP2B* genes [50] probably as an homeostatic mechanism.

The IL-17 family is important for the production of antimicrobial peptides. Among the cells that can respond to the stimulus in skin are keratinocytes, mast cells, and neutrophils [37]. Included as a subgroup is the S100, composed by 21 different types [38]. S100A7 gene encodes psoriasin [39] and we found that SNP rs12049559, was accessible in the NHEK and HAEpiC cell line, and alters the ELF1 transcription factor. This SNP was not analyzed by GTEx project (Supplementary Figure S1). In murine models, ELF1 regulates the development and activation of different cells like T, B, macrophages as well as gene regulation of cells from epithelium [40].

We included also inflammatory cytokines coding genes *TNF* and *IFNG* that are known to be increased in psoriatic lesions [41]. In *TNF* gene we found that rs361525 and rs1800629 alters TFBS related to the epithelial-mesenchymal transition (EMT). It is known that Msx2 play a role in promoting EMT, inducing changes in cell morphology and disruption of cell-cell contacts in transfected cells [42], c-JUN also promotes a migratory phenotype in melanoma cells [43]. The set of β-catenin/TCF4 with aberrant activation is a key factor in the genesis of many cancers, since they induce EMT through its direct target ZEB1 [44]. On the other hand, the loss and the silencing of PRRX1 is also capable of promoting EMT, leading cells to acquire a mesenchymal phenotype [45]. TNFα and various NF-κB activators induce, in stages of chronic inflammation, the expression of Twist1 leading to an invasion of cancer and angiogenesis, associated with EMT [46]. These TFs that we found could be playing a role in the development of changes in epithelium in psoriasis. Other groups have reported the participation of TNF in normal and psoriatic keratinocytes, with a clear increase in K16 epithelial marker and FN mesenchymal marker, although they conclude that this participation could be pluripotent, as it can also decrease K10 and Slug, epithelial and mesenchymal markers respectively [47]. Regarding *IFNG* gene, we found no accessible chromatin in the promoters of the analyzed cell lines.

The remaining genes with no predicted rSNP by SNPClinic, may not have transcriptional relevance in psoriasis but instead may be mainly regulated by metal ions (calprotectin), dimerization and abundance (calprotectin and NFKB1 as heterodimer of NF-kB), homotypic redundance (STAT1 and NFKB1 transcription factors) or an altered alternative layer of gene regulation as Pathogen-Associated Molecular Pattern-dependent expression (defensins, remaining interleukins and their receptors).

Even when the SNPClinic v.1.0 software is a powerful tool that aid for a better understanding of the molecular mechanism between rSNPs and gene expression, the relationship of rSNPs/gene expression with pathogenicity is far more complex and is a topic that our group is very interested to elucidate and to predict more accurately. We pretend to apply this “pathogenicity score” to e.g. polygenic risk scores (Prado Montes de Oca et al., in process) and pharmacogenetics (Chávez Álvarez and Prado Montes de Oca, in process). Prediction of pathogenicity based on both genetic and non-genetic variants must include many other features as non-coding RNAs, transcription factor grammar, protein synthesis and degradation rates, proteomics (proteins present in a sample at a given time and condition), 3D interactions, metabolome and other relevant data as well. The above mentioned massive data must be wisely weighted and integrated with a functional genomics approach, which main aim is to understand the dynamic expression of gene products in a specific context (https://www.ebi.ac.uk/training-beta/online/courses/functional-genomics-i-introduction-and-design/what-is-functional-genomics/) to develop a more rational model to link both genotype plus other personalized data in order to predict in a most trustable way the prognosis of clinic phenotypes in psoriasis. These *in silico* results are interesting and have strong *in vitro* support since SNPclinic uses ENCODE and JASPAR databases as inputs (which are supported by an extensive collection of *in vitro* experiments). In spite of its limitations, SNPClinic results are a more rational starting point that can propose novel *in vitro* and *in vivo* experiments as well as research and development projects which results will allow to validate novel drugs targets and companion diagnostics. Tools like SNPClinic software v.1.0 open new avenues to a comprehensive personalized medicine for psoriasis patients.

## 4 Conclusions

Results obtained with traditional available software is very limited in the prediction of rSNPs with a cell line/tissue- and disease-specific approach. We found six rSNPs in five genes that may explain hallmark processes in psoriasis as hyperproliferation of keratinocytes*,* T-cell polarization and epithelial-mesenchymal transition. These six putative rSNPs will aid in the design and interpret rational *in vitro* and *in vivo* experiments in psoriasis research for the quest of novel drug targets (as the highest ranked transcription factors–absolute value of ≥10-by the FIF parameter of SNPClinic - their altered binding sites when a common SNP is present, cathelicidin and psoriasin) and their companion diagnostics (rSNPs). Furthermore, the putative four rSNPs involved in *TNF, IL17C* and *IL1*7RA regulation that we found, could be validated in the clinic as companion diagnostics/pharmacogenetics assays for prescribed drugs for current psoriasis therapy as brodalumab, secukinumab, ixekizumab, infliximab, certolizumab, etanercept, golimumab and adalimumab.

## Author statement

Andrea Virginia Ruiz Ramírez: Investigation, interpretation of data, Visualization, Writing – original draft preparation; Adolfo Flores Saiffe Farías: Software, Investigation; Rocío del Carmen Chávez Álvarez: Software, Investigation; Ernesto Prado Montes de Oca: Conceptualization, design, Funding acquisition, Resources, interpretation of data, writing-review, editing & revision.

## Declaration of competing interest

EPM has a granted patent for a small molecule that inhibits *CAMP* gene (cathelicidin LL-37) transcription.
