# Supplementary material for: Predicted regulatory SNPs reveal potential drug targets and novel companion diagnostics in psoriasis
Source: J Transl Autoimmun. 2021 Apr 5;4:100096. doi: 10.1016/j.jtauto.2021.100096 (PMC8060581; doi:10.1016/j.jtauto.2021.100096)
Supplement: Multimedia component 1 [file mmc1.docx]

SUPPLEMENTARY MATERIAL


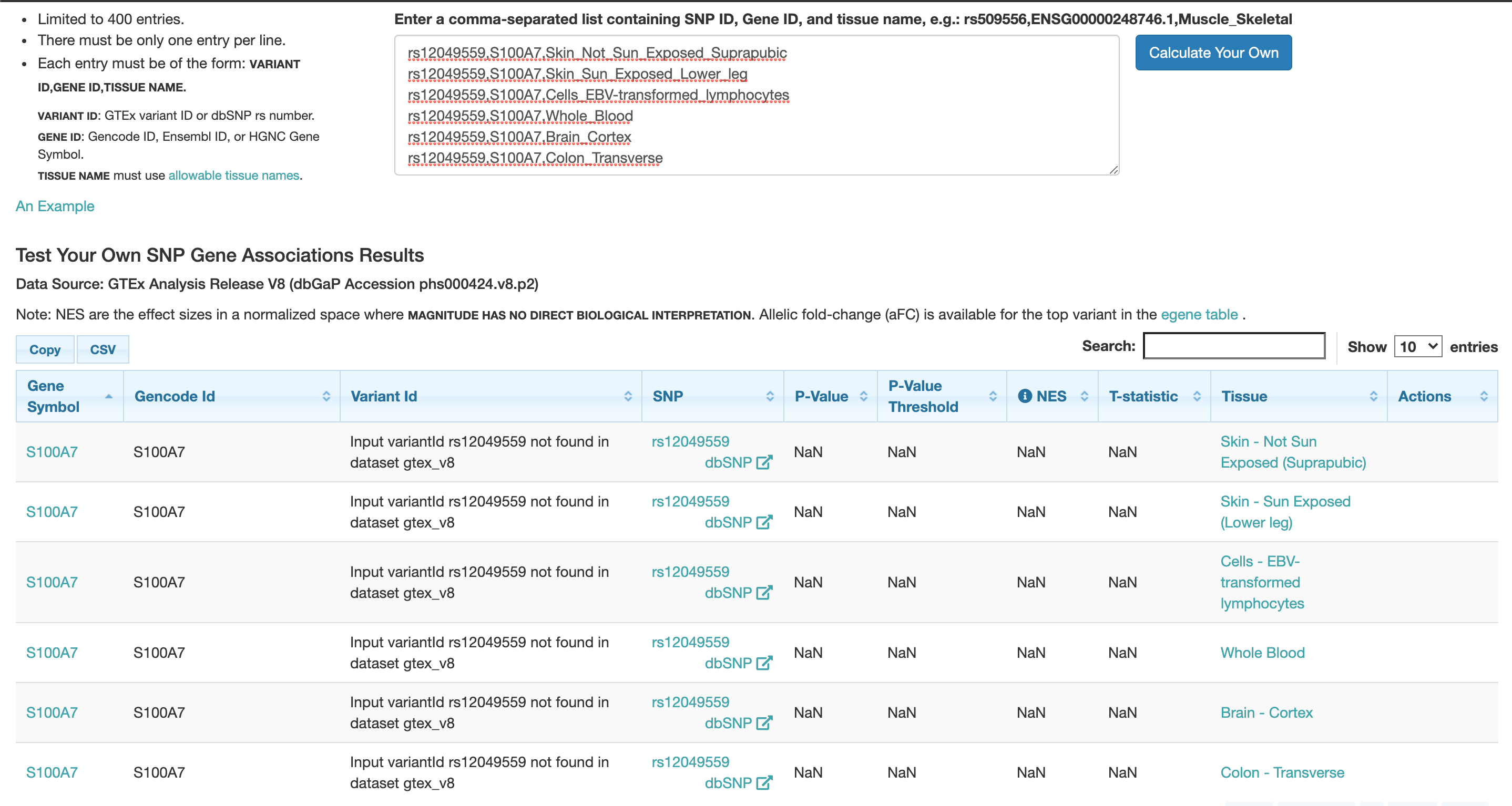


Figure S1. The rSNP rs12049559 in *S100A7* gene (psoriasin) is not available as eQTL in GTEx database.
